# Bond‐Competition‐Driven Enhancement of Surface Basicity and Interfacial Interaction to Boost Photocatalytic Syngas Production

**DOI:** 10.1002/advs.76890

**Published:** 2026-08-03

**Authors:** Dong Wook Lee, Wenjing Dong, Nam Hee Kwon, Taehoon Kim, In Young Kim, Yun Kyung Jo, Xiaoyan Jin, Ajayan Vinu, Yufei Zhao, Seong‐Ju Hwang

**Affiliations:** ^1^ Department of Materials Science and Engineering College of Engineering Yonsei University Seoul Republic of Korea; ^2^ State Key Laboratory of Chemical Resource Engineering Beijing University of Chemical Technology Beijing People's Republic of China; ^3^ Department of Chemistry and Nanoscience Ewha Womans University Seoul Republic of Korea; ^4^ Department of Applied Chemistry University of Seoul Seoul Republic of Korea; ^5^ Global Innovative Center for Advanced Nanomaterials The University of Newcastle Callaghan NSW Australia; ^6^ Department of Battery Engineering Yonsei University Seoul Republic of Korea

**Keywords:** bond competition, CO_2_ adsorption, CO_2_ reduction, photocatalytic syngas production, sulfur doping, surface basicity control

## Abstract

The adsorption and photocatalytic conversion of CO_2_ molecules to mitigate atmospheric greenhouse gas concentrations and manufacture value‐added chemicals require efficient CO_2_ reduction reaction catalysts. In this study, a surface bond competition approach was developed to obtain high‐performance CO_2_ adsorbents and syngas production photocatalysts via the sulfurization‐driven enhancement of surface basicity and interfacial interaction. The heat treatment of Mg–Al‐layered double hydroxide nanosheets under a flow of CS_2_ yielded sulfur‐doped MgO/MgAl_2_O_4_S_x_ nanosheets. The sulfur‐doping‐induced enhancement of surface basicity originated from the increased electron density on oxygen through competition with covalent metal–sulfur bonds, substantially enhancing the CO_2_ adsorptivity. The sulfur‐doped MgO/MgAl_2_O_4_S_x_ nanosheets acted as effective hybridization matrices for ZnIn_2_S_4_ nanoplates, boosting their activity for photocatalytic syngas production (i.e., ≈3.3 mmol g^−1^ h^−1^ with the ratio of CO/H_2_ = 2.2). Density functional theory calculations revealed that hybridization with MgO/MgAl_2_O_4_S_x_ nanosheets was effective in lowering both the adsorption energy of CO_2_ and the energy barrier for the conversion of *COOH to *CO. Systematic in situ spectroscopic investigations highlighted that the hybridization with MgO/MgAl_2_O_4_S_x_ enhanced Lewis acid−base interaction between ZnIn_2_S_4_ and absorbed CO_2_, and the contribution of associative pathways, which were attributed to sulfur‐doping‐assisted reinforcement in interfacial electronic coupling between hybridized components.

## Graphical abstract

A synergetic surface bond competition−hybridization approach was developed to synthesize high‐performance CO_2_ adsorbents and syngas production photocatalysts via the sulfurization‐driven control of surface basicity and interfacial coupling. The ZnIn_2_S_4_−sulfur‐doped MgO/MgAl_2_O_4_ nanocomposites showed outstanding photocatalytic efficiency for production of CO and syngas, which could be attributed to enhanced surface adsorptivity, increased surface reactivity toward CO_2_, and reinforced interfacial interaction.

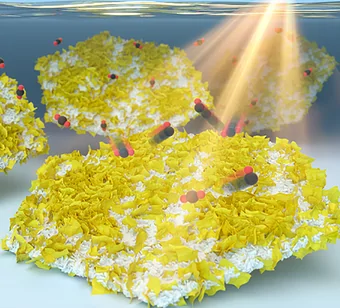

## 1 Introduction

The increasing threat of global climate change has sparked tremendous interest in the sequestration of greenhouse gases [1, 2, 3, 4]. The adsorption and subsequent photocatalytic conversion of CO_2_ molecules can provide an effective and economically feasible procedure for mitigating atmospheric greenhouse gas concentrations and manufacturing value‐added chemicals [5, 6, 7, 8, 9]. To achieve an efficient CO_2_ reduction reaction (CO_2_RR), it is essential to simultaneously optimize the surface adsorption properties and photocatalytic activities of the catalyst materials. In this context, hybridization between gas adsorbents and reduction‐active photocatalysts offers valuable opportunities for the development of high‐performance photocatalysts for CO_2_RR as well as hydrogen evolution reaction (HER) [10, 11]. To date, various microporous materials, such as zeolite‐based materials and metal–organic frameworks, have been exploited as efficient CO_2_ adsorbents [12, 13, 14, 15]. However, the nanoscale hybridization of these microporous materials with photocatalysts results in pore blockage, leading to significant degradation of the CO_2_ adsorptivity. Alternatively, basic inorganic solids, including alkaline earth metal oxides, can serve as efficient hybridization matrices for photocatalysts because their chemisorption‐dominant mechanism is less sensitive to surface porosity [16, 17, 18]. One of the most relevant adsorbents for hybridization with photocatalysts is the layered double oxide (LDO), which is prepared by the heat treatment of a layered double hydroxide (LDH) at elevated temperatures [19]. Considering that the chemisorption of CO_2_ depends strongly on the surface basicity of metal oxides [20], the high compositional tunability of the LDH precursors allows the optimization of CO_2_ adsorption in calcined LDOs via doping‐mediated control of surface basicity [21]. As illustrated in Figure 1a, sulfur doping of the LDO lattice enhances the ionicity of neighboring metal–oxygen bonds via competition with highly covalent metal–sulfur bonds [22], consequently boosting CO_2_ adsorptivity due to an increased electron density on oxygen. Accordingly, the hybridization of reduction‐active photocatalysts with sulfur‐doped LDOs was expected to promote the photocatalytic reduction of CO_2_ and production of syngas (CO + H_2_). The photocatalytic syngas production has garnered significant attention because of its high economic value because the syngas is a crucial feedstock for various value‐added organic chemicals [23, 24]. For example, the Fischer−Tropsch synthesis (FTS) utilizing syngas to manufacture synthetic fuels offered a sustainable closed‐carbon alternative to conventional petroleum processing [25]. CO‐rich syngas (CO/H_2_> 1), achievable by enhanced CO_2_RR selectivity, is especially advantageous for ecofriendly biological fermentation for alcohol and fatty acids synthesis, direct dimethyl ether synthesis, and the FTS coupled with the water−gas shift reaction [26, 27, 28, 29, 30]. The co‐production of the syngas mixture is also economically favorable for the aforementioned industrial applications compared to direct production of pure CO or H_2_ because it circumvents the requirement for deliberate mixing of the separated monomers. Additionally, sulfur doping of the LDO can enhance chemical interactions with metal‐sulfide‐based photocatalysts via the optimization of soft−hard Lewis acid−base interactions. Despite the expected advantages of surface basicity control and interfacial interactions between the adsorbent and photocatalyst, no previous studies have focused on optimizing CO_2_ absorptivity and syngas production ability via a sulfur‐doping‐assisted bond competition approach.

**FIGURE 1:**
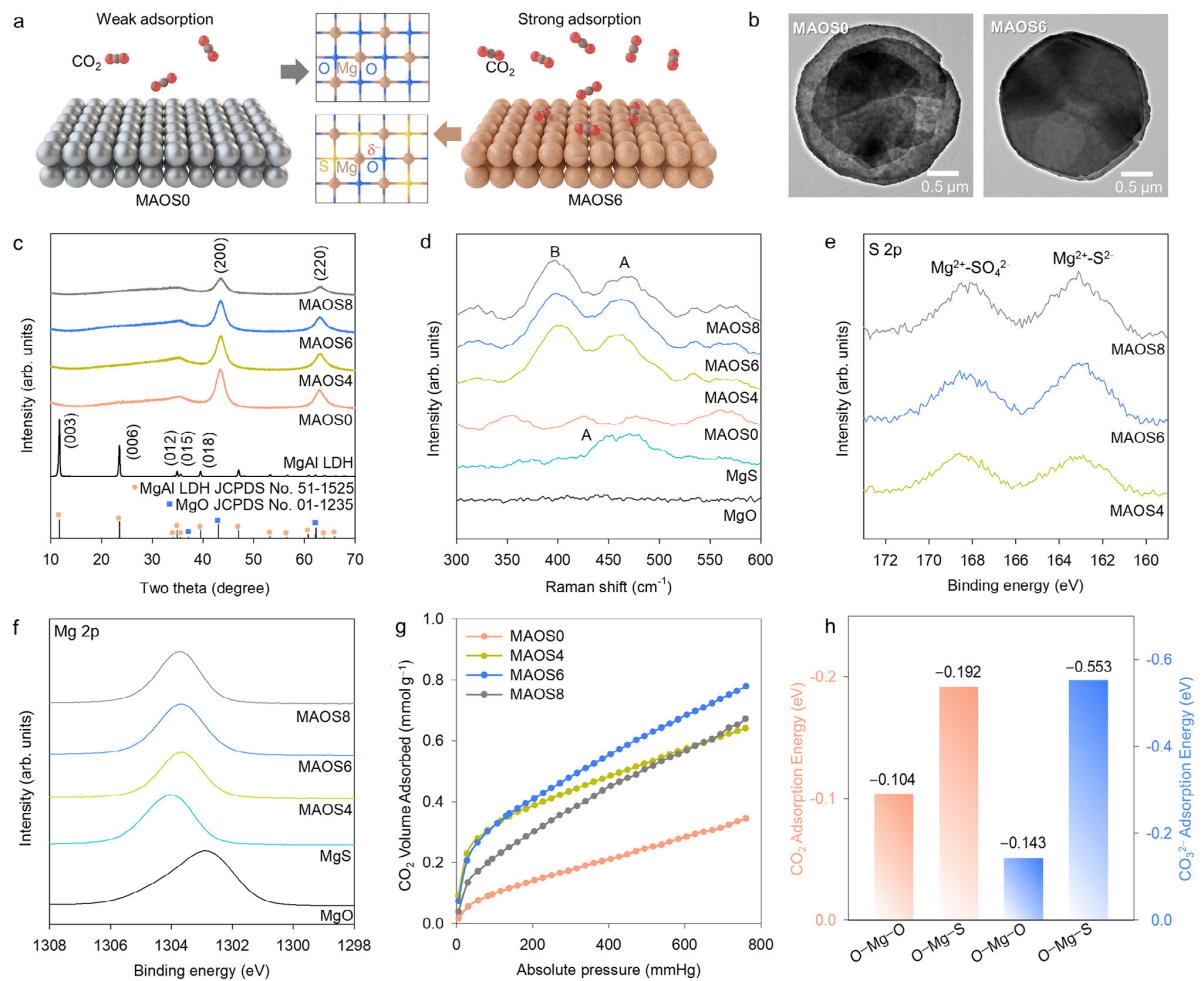
a) Schematic illustration of the enhanced CO_2_ adsorption induced by the bond competition in the sulfur‐doped MgO/MgAl_2_O_4_S_x_. b) TEM, c) XRD, d) micro‐Raman, e) S 2p XPS, f) Mg 2p XPS, and g) CO_2_ adsorption isotherms. h) DFT calculated adsorption energies of CO_2_ and CO_3_^2−^ on O−Mg−O and O−Mg−S.

In this study, a surface bond competition strategy is developed to explore highly efficient CO_2_ adsorbents and photocatalysts for syngas production. Specifically, this approach is based on the sulfur‐doping‐induced enhancement not only for the surface basicity of LDO nanosheets but also for their interfacial interaction with reduction‐active ZnIn_2_S_4_ (i.e., ZIS) photocatalyst. The effects of sulfur doping on the surface basicity, CO_2_ adsorption energy, and interfacial chemical bond formation of the MgO/MgAl_2_O_4_S_x_ (i.e., MAOS) nanosheets are investigated using a series of experimental and density functional theory (DFT) analyses. Additionally, modifications to the chemical bonding nature, interfacial charge transfer, and surface reactivity of the ZIS–MAOS (ZM) nanocomposites during the CO_2_RR are systematically examined using combined in situ spectroscopic analyses to elucidate the underlying mechanism responsible for the surface‐basicity‐control approach.

## 2 Results and Discussion

### 2.1 Effects of Sulfur Doping on the Crystal Structure, Morphology, and Surface Nature of the MAOS Nanosheets

The sulfur‐doped MAOS nanosheets were synthesized by heat treatment of Mg–Al‐LDH nanosheets at 450°C under a flow of CS_2_ gas [31]. Notably, the lower sulfurization power of CS_2_ compared with that of the widely used H_2_S gas allowed the effective introduction of sulfur doping onto the surface [32]. The overall reaction between CS_2_ and MgO/MgAl_2_O_4_, i.e., MgO/MgAl_2_O_4_ + CS_2_ → MgO/MgAl_2_O_4_(S) + COS, was exothermic [33], since the formation of C═O bond at the expense of a C═S bond overcompensated for the energy required for the substitution of an O atom of MgO with a S atom (energy change values: −0.27 eV for terrace site, −1.45 eV for step site, −1.74 eV for edge site, and −1.49 eV for corner site of MgO). To control the degree of sulfur doping, several volumes of CS_2_ (0, 0,4, 0.6, and 0.8 mL) were employed per 200 mg of the precursor Mg–Al‐LDH nanosheets; the resulting materials were denoted MAOS0, MAOS4, MAOS6, and MAOS8, respectively. The sulfur doping induced a distinct color change to yellow from white sulfur‐free MAOS0, which intensified with increased CS_2_ content. Transmission electron microscopy (TEM) confirmed the retention of the 2D nanosheet morphology of the MAOS materials after sulfurization (Figure 1b). Calcination under CS_2_ gas flow increased the surface roughness, further reflecting surface modification through the formation of dangling metal–sulfur bonds. As detailed in Table S1, inductively coupled plasma–optical emission spectroscopy (ICP–OES) revealed a gradual increase of the S/(Mg+Al) ratios of MAOS nanosheets upon increased CS_2_ volumes, demonstrating the controllability of sulfur composition. All MAOS nanosheets exhibited typical X‐ray diffraction (XRD) peaks corresponding to the rocksalt‐structured MgO phase (Figure 1c). Although the heat treatment of Mg–Al‐LDH is known to produce nanocomposites consisting of MgO and MgAl_2_O_4_ phases [34], Bragg reflections corresponding to the MgAl_2_O_4_ phase were not clearly discernible, indicating poor crystallinity. Owing to the higher ionicity of the Mg–O bond, the MgO component was primarily responsible for promoting CO_2_ adsorption on the MAOS surface [35]. The sulfur‐doped MAOS did not display any XRD peaks related to the metal sulfide phases, indicating the absence of metal sulfide phase separation. Furthermore, least squares fitting analysis demonstrated that CS_2_ treatment did not cause any significant change in the unit cell parameter of MgO (a = 4.176 Å), although marked differences in the ionic sizes of O^2−^ and S^2−^ were evident [36]. The observed negligible alteration in the lattice parameter confirms the formation of dangling metal–sulfur bonds at the surfaces and/or grain boundaries, rather than the substitution of sulfur for lattice oxygen species. Simultaneously, sulfur doping caused a gradual depression and broadening of the MAOS‐related peaks, indicating that the overgrowth of the MgO domain was prevented via the formation of dangling Mg−S bonds at the grain boundaries. This was confirmed by the calculation of primary MgO particle size based on the Scherrer equation, showing the sulfurization‐induced size reduction (i.e., 4.63 nm for MAOS0, 4.43 nm for MAOS4, 4.38 nm for MAOS6, and 4.00 nm for MAOS8). X‐ray photoelectron spectroscopy (XPS)‐depth profiling using Ar^+^‐ion etching was conducted to define the precise doping sites of sulfur atoms. As presented in Figure S1, Ar‐ion etching with a high power did not cause any significant intensity change in sulfur‐related XPS signals, clearly demonstrating that the sulfur atoms were stabilized not only on the sample surface but also on the grain boundary inside the sample. This could be interpreted as a result of very thin 2D crystal shape of MAOS, allowing for the efficient diffusion of sulfur dopants inside the bulk state.

The formation of metal–sulfur bonds in the MAOS nanosheets was confirmed by micro‐Raman spectroscopy and XPS. As depicted in Figure 1d, in contrast to MAOS0, the sulfur‐doped MAOS nanosheets displayed two Mg–S bond‐related Raman peaks (A and B) in the region of ≈350–520 cm^−1^, resembling MgS. Splitting of these phonon lines suggested Mg–S bond diversification due to bond competition with neighboring Mg–O bonds. The S 2p XPS spectrum of the MAOS nanosheets in Figure 1e exhibited an intense broad peak corresponding to the sulfide ion (S^2−^) at a binding energy (BE) of ≈162.5 eV, confirming the introduction of Mg–S bonds. Although an additional XPS peak originating from sulfate ions (SO_4_^2−^) was discernible at ≈169.0 eV [37], the Raman spectra of the MAOS nanosheets did not show any sulfate‐related phonon lines [38]. Based on the surface sensitivity of XPS, the observed discrepancy suggests atmospheric oxidation of the sulfur atoms present on the MAOS surfaces. The Mg 2p XPS data of the MAOS nanosheets in Figure 1f displayed a strong peak corresponding to the divalent Mg^2+^ ion at ≈1303.8 eV positioned between the reference MgO and MgS peaks, supporting the partial sulfurization. The Mg K‐edge X‐ray absorption near‐edge structure (XANES) spectra (Figure S2a) revealed that MAOS0 exhibited several intense peaks (A, B, and C) at ≈1312, ≈1318, and ≈1321 eV, respectively. Peaks A and C are characteristic of multiple scattering from the outermost coordination shells [39, 40, 41], whereas peak B originates from dipole‐allowed 1s → 3p transition. Sulfur doping notably enhanced the spectral weights of peaks A and B (Figure S2b) like MgS, confirming the introduction of Mg–S bonds.

### 2.2 Sulfur‐Doping‐Induced Improvement of the MAOS CO_2_ Adsorption Capability

The profound impact of sulfur doping on the CO_2_ adsorption behavior was confirmed by gas adsorption–desorption isotherm measurements. As shown in Figure S3, all MAOS nanosheets commonly displayed the Brunauer–Deming–Deming–Teller (BDDT) type IV isotherm and H3‐type hysteresis [42], indicating the formation of house‐of‐cards‐type aggregates of plate‐like crystallites [43]. Based on the Brunauer–Emmett–Teller (BET) equation, the surface areas were determined to be 145, 117, 94, and 81 m^2^ g^−1^ for MAOS0, MAOS4, MAOS6, and MAOS8, respectively (Table S2). This result indicates that heat treatment under CS_2_ flow reduced the MAOS surface area owing to mesopore collapse, supported by the mesopore volumes calculated using the Barrett–Joyner–Halenda (BJH) method [44]. However, the CO_2_ adsorption capability of the MAOS nanosheets was greatly enhanced by sulfur doping, emphasizing the benefits of metal–sulfur bond introduction (Figure 1g). Specifically, at pressures <100 mmHg, the CO_2_ adsorption isotherms of the sulfur‐doped MAOS nanosheets exhibited steeper initial slopes corresponding to the chemisorption of CO_2_ compared to MAOS0 [45], emphasizing enhanced CO_2_ chemisorption via increased surface basicity. Similarly, at higher pressures of >100 mmHg, a steeper slope occurred for the heavily sulfur‐doped MAOS6 and MAOS8, reflecting the surface‐roughening‐assisted enhancement of CO_2_ physisorption. The MAOS nanosheets also demonstrated excellent CO_2_ adsorptivity at 0°C, exhibiting their exceptional functionality as CO_2_ adsorbents over a wide temperature range (Figure S4). Among the MAOS nanosheets, MAOS6 boasted the largest gravimetric CO_2_ adsorptivity (0.779 mmol g^−1^), confirming the optimal sulfur‐doping efficiency for this material (Table S2). Beyond this optimum, the gravimetric CO_2_ adsorptivity decreased, possibly because of a notable reduction in the surface area. Contrastingly, the surface‐area‐normalized areal CO_2_ adsorptivity of the MAOS nanosheets continuously improved with increasing sulfur content, emphasizing the high efficiency of surface doping in enhancing the CO_2_ adsorption efficiency. As summarized in Table S3, the MAOS6 nanosheet outperforms recently reported Mg–Al‐LDO‐related materials, underscoring the validity of the basicity control approach for CO_2_ adsorptivity.

The sulfurization‐driven enhancement of the surface basicity was evidenced using the benzoic acid titration method [46]. As detailed in Table S4, the sulfur‐doped MAOS nanosheets exhibited higher basic site densities than MAOS0. Since the Mg–S bond is more covalent than the Mg–O bond, the introduction of Mg–S bonds lowers the covalency of the Mg–O bond. The resulting increase in the electron density of the oxygen ions enhanced surface basicity and promoted CO_2_ chemisorption, as confirmed by differential charge density analysis and Bader charge analysis (Figure S5 and Table S5). The profound impact of sulfur doping on the bonding character and CO_2_ reactivity was also theoretically demonstrated through DFT calculations. Specifically, sulfur doping into the MgO crystal elongated the Mg–O bond from 2.106 to 2.114 Å, confirming a reduced covalency (Figure S6) [47]. Considering that CO_2_ adsorptions usually occur on the MgO surface, two possible adsorption modes were taken into account: adsorption of the oxygen atom of CO_2_ on the Mg sites (Figure S7a,b), and the vertical adsorption of carbonate ions (CO_3_^2−^) on the oxygen sites of MgO (Figure S7c,d) [48]. As presented in Figure 1h, MgO exhibited adsorption energies of 0.104 eV for adsorbed CO_2_ and 0.143 eV for CO_3_^2−^ (absolute values). Following sulfur doping on the MgO surface, the adsorption energies of CO_2_ and CO_3_^2−^ increased to 0.192 and 0.553 eV, respectively, thereby supporting the sulfur‐doping‐driven enhancement of CO_2_ adsorption efficiency.

### 2.3 Intimate Interfacial Interactions within the Hybridized MAOS/ZIS

The basicity‐optimized MAOS6 nanosheets were employed as hybridization matrices for the ZIS nanoplates by ball‐milling (Figure 2a and Figure S8). To probe the effect of the MAOS content, MAOS6/ZIS weight ratios of 10, 25, and 50 wt% were employed and denoted as ZM10, ZM25, and ZM50, respectively. The TEM data in Figure 2b demonstrated that the ZIS nanoplates were immobilized on the surfaces of larger 2D MAOS6 nanosheets with suppressed aggregation. This is in stark contrast to the pristine ZIS material exhibiting a micro‐flower morphology comprising densely aggregated nanoplates (Figure S9), underscoring the enhanced chemical interaction between sulfur‐doped MAOS6 and ZIS materials. The EDS‐elemental mapping images in Figure 2b showed a homogenous distribution of Mg, Al, Zn, In, and S throughout the ZM25 nanocomposite, substantiating uniform hybridization between MAOS and ZIS. The selected area electron diffraction (SAED) pattern in Figure 2c and high resolution transmission electron microscopy (HRTEM) image in Figure S10 provided additional convincing evidence for the homogeneous hybridization between ZIS and MAOS. Specifically, lattice fringes corresponding to the (006) planes of ZIS and the (200) planes of MAOS were clearly discernible for the ZM25 nanocomposite. Powder XRD analysis (Figure 2d) confirmed that all ZM nanocomposites showed typical Bragg reflections corresponding to the hexagonal ZIS phase (JCPDS No. 65–2023) with no visible MAOS‐related peaks, demonstrating the homogeneous dispersion of MAOS6 without phase separation. The use of ethanol as a coolant during the ball‐milling procedure effectively minimized the phase transition from hexagonal to cubic ZIS. The N_2_ adsorption–desorption isotherms exhibited the decrease of BET surface area reduction from 127 m^2^ g^−1^ for ZIS to 115, 101, and 98 m^2^ g^−1^ for the hybridized ZM10, ZM25, and ZM50, respectively, attributed to the partial blocking of the ZIS mesopores by MAOS after hybridization (Figure S11).

**FIGURE 2:**
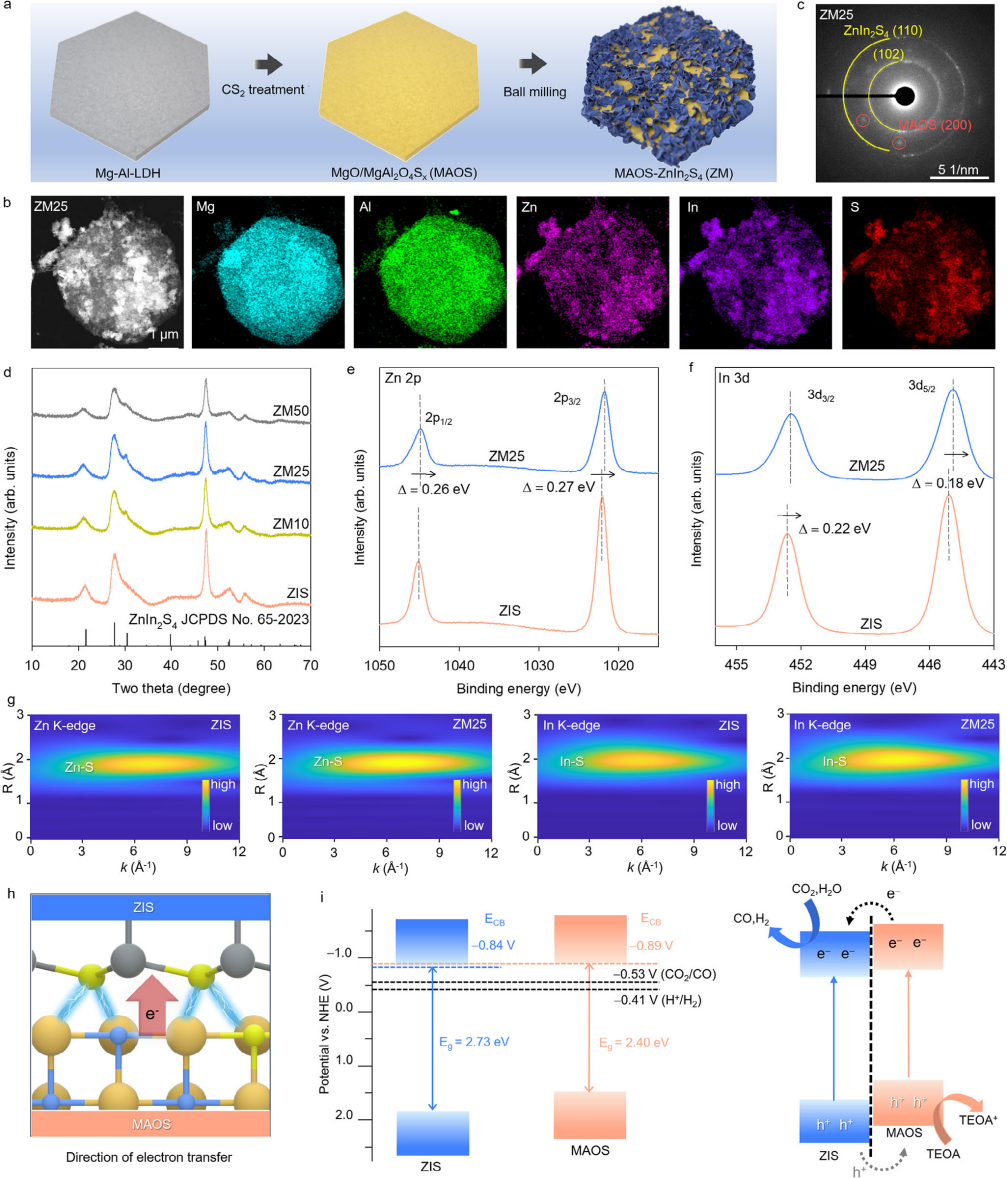
a) Schematic illustration for the synthesis of the ZM nanocomposite. b) TEM−EDS‐elemental map, c) SAED, d) XRD, e) Zn 2p XPS, f) In 3d XPS, and g) WT‐EXAFS data. h) Schematic illustration for the interfacial chemical interaction between ZIS and MAOS. i) Electronic band structures of ZIS and MAOS6 with the electron transfer scheme for the heterojunction in ZM25 nanocomposite.

The distinct effects of interfacial interaction with MAOS6 on the local geometry and electronic structure of ZIS were verified using XPS and EXAFS. Compared with ZIS, the ZM25 nanocomposite displayed slightly lower BEs for the Zn 2p and In 3d XPS peaks (Figure 2e,f), suggesting that the Zn and In valence states were reduced due to charge transfer from MAOS to ZIS. This finding emphasizes the increase in the electron density and Lewis basicity of ZIS by the interfacial electronic coupling with sulfur‐doped MAOS6.

Extended X‐ray absorption fine structure (EXAFS) analysis substantiated the sulfur‐doping‐assisted formation of interfacial bonds between ZIS and MAOS. The Zn K‐edge EXAFS data in Figure S12 demonstrated that both the ZIS and ZM nanocomposites displayed an intense Fourier transform (FT) peak originating from the Zn–S bonding pair at ≈1.9 Å with sulfur‐doping‐driven increase of peak intensity. The EXAFS fitting analysis revealed that an increase of sulfur content in the ZM nanocomposites led to a progressive increase in the coordination number for the Zn–S bonding pair, highlighting the sulfur‐doping‐enhanced formation of interfacial Zn–S coordination bonds between ZIS and sulfur‐doped MAOS (Table S6). Likewise, the In K‐edge EXAFS spectra of ZIS and ZM nanocomposites exhibited an In–S‐related FT peak at ≈2.0 Å and increased coordination number for the In–S bonding pair with increased sulfur content, corroborating the formation of interfacial In–S bonds (Figure S13 and Table S7). In addition, Zn K‐edge and In K‐edge EXAFS analyses for ZM nanocomposites hybridized with different amounts of sulfur‐doped MAOS0, MAOS4, MAOS6, and MAOS8 revealed that an increased sulfur doping enhanced the FT peaks corresponding to Zn–S and In–S bonding pairs (Figure S14), reflecting the promotion of interfacial bond formation between Zn/In in ZIS and S in MAOS. This can be regarded as convincing evidence for the sulfur‐doping‐induced enhancement of soft Lewis acid−base interaction between ZIS and MAOS. The ionocovalent nature of interfacial bonds between ZIS and MAOS was further confirmed by the fact that experimentally determined Zn−S and In−S bond distances lied between the sums of covalent and ionic radii of metal and sulfur (Tables S8−S10) [49].

This conclusion was further supported by Zn K‐edge and In K‐edge wavelet transform (WT)‐EXAFS analyses, in which the most intense features at both edges were stronger for ZM25 than for ZIS, indicating an enhanced metal coordination environment in ZM25 (Figure 2g). The enhanced interfacial bond formation between ZIS and MAOS6 corroborated by EXAFS results can be attributed to the enhanced soft−hard Lewis acid−base interaction. Namely, the sulfur doping of MAOS increased its softness, reinforcing interfacial coordination with the soft ZIS lattice. Importantly, the resultant interfacial bonds can promote electronic coupling between these hybridized species via the inner‐sphere mechanism (Figure 2h).

To understand the effect of hybridization on photocatalytic activity, the band structure of the ZM nanocomposite was experimentally determined. As presented in the Tauc plots (Figure S15), the bandgap energies of ZIS and MAOS6 were determined to be 2.73 and 2.40 eV, respectively. A positive slope was commonly discernible in the Mott−Schottky plots of ZIS and MAOS6 (Figure S16), indicating their n‐type semiconducting natures [50]. Considering that the conduction band minimum is usually positioned 0.2 eV below the flat‐band potential for n‐type semiconductors [51], the conduction band minima for ZIS and MAOS6 were calculated to be −0.84 and −0.89 V (vs NHE), respectively, which are relevant for the reduction of adsorbed CO_2_ to CO (−0.53 V vs. NHE) and H^+^ to H_2_ (−0.41 V vs NHE) [52]. As plotted in Figure 2i, the valence band (VB) and conduction band (CB) of MAOS6 showed higher energies than those of ZIS. Thus, the excited electrons in the CB of MAOS6 could be migrated to the CB of ZIS, whereas the holes in the VB of ZIS could move to the VB of MAOS6. Such interfacial charge transfer allowed efficient charge separation and the extension of electron−hole lifetimes, both beneficial in improving the photocatalytic activity [53].

### 2.4 Improved Photocatalytic CO_2_RR Performances of the ZM Nanocomposites

The benefits of hybridization of sulfur‐doped MAOS with ZIS for photocatalytic performance were verified by employing ZM nanocomposites as CO_2_RR photocatalysts in a liquid mixture system with a 10% CO_2_ concentration. Notably, ZM nanocomposites demonstrated superior photocatalytic CO_2_RR activity and selectivity over ZIS (Figure 3a,b), eventually resulting in optimized photocatalytic syngas production efficiency (Figure 3c). The ^1^H nuclear magnetic resonance spectroscopy also detected the formation of formate ions, although the amount of formate ions was insufficient for subsequent product separation and purification (Figure S17) [54, 55]. Among the synthesized samples, the composition‐optimized ZM25 nanocomposite delivered the highest syngas production rate of ≈3.3 mmol g_cat_^−1^ h^−1^ with a 2.2 ratio of CO/H_2_ (Figure 3c), surpassing recently reported heterogeneous photocatalysts (Figure 3d and Table S11) [56, 57, 58, 59, 60, 61, 62, 63]. Additionally, ZM25 displayed excellent retention of photocatalytic activity over ten consecutive cycles, highlighting its robust photostability (Figure S18). The excellent stability of ZM25 was further confirmed by no significant changes in its XRD and XPS data after the reaction with negligible degrees of metal‐ and sulfide‐ion leaching (Figures S19 and S20). Furthermore, the role of MAOS6 hybridization in boosting photocatalytic activity was corroborated by the larger photocurrents observed for the ZM nanocomposites compared to pristine ZIS (Figure S21). Closer inspection revealed a slower decay rate, highlighting the suppression of charge recombination upon hybridization. The crucial role of hybridization with sulfur‐doped MAOS in the high CO_2_RR activity of ZM25 was further corroborated by several control experiments. Specifically, MAOS6 alone displayed no catalytic activity (Figure S22), confirming that it solely acts as a hybridization matrix. Additionally, no CO or H_2_ gases were generated in the absence of ZM25 or visible light illumination, demonstrating that the photocatalytic reduction of CO_2_ was driven by ZM25. Moreover, in a CO_2_‐free N_2_ atmosphere, the visible light irradiation generated H_2_ gas only. Thus, the ZM25 material could be used for the generation of pure H_2_ under CO_2_‐free condition (Figure S22). In addition, gas chromatography−mass spectroscopy analysis with isotope ^13^CO_2_ confirmed that the produced CO molecules originated from the reduction of atmospheric CO_2_ (Figure S23). To verify the pivotal role of sulfur doping in enhancing CO_2_RR activity, ZIS was hybridized with 25 wt% MAOS0 to prepare ZM025. While the XRD results confirmed successful hybridization (Figure S24), ZM025 displayed substantially poorer CO_2_RR activity (813 µmol g_cat_^−1^ h^−1^) (Figure S25), underscoring the benefits of sulfur‐doping in photocatalytic activity enhancement.

**FIGURE 3:**
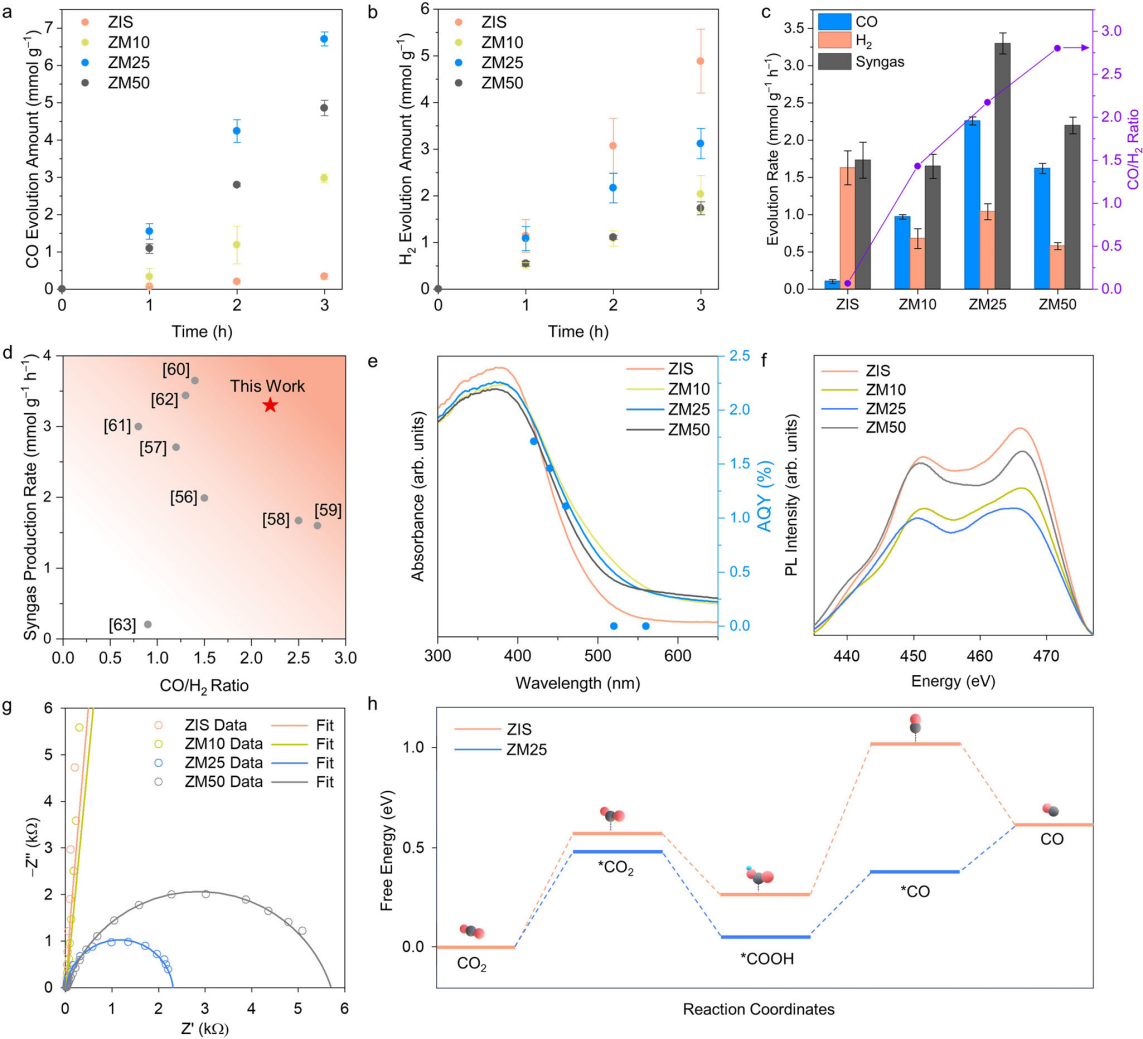
Visible‐light‐driven photocatalytic evolution rates of a) CO and b) H_2_. c) Gas evolution rates and CO/H_2_ ratio. d) Comparison plot of the syngas production performance of ZM25 to those of recently published photocatalysts. e) Diffuse‐reflectance UV−vis and AQY, f) PL, g) EIS, and h) DFT‐calculated reaction coordinates for ZIS and ZM25.

As plotted in the diffuse reflectance UV−vis spectra, ZM nanocomposites exhibited red‐shifts of optical absorption edges with respect to pristine ZIS (Figure 3e). Apparent quantum yields (AQY) of ZM25 were measured at several wavelengths of monochromatic light, and the trend was found to align with the absorption behavior (Figure 3e). The Tauc plots in Figure S26 demonstrated decreased optical band gaps for ZM nanocomposites compared to ZIS (2.73 eV), indicating a hybridization‐driven increase in visible‐light absorptivity. Additionally, the ZM nanocomposites displayed lower photoluminescence (PL) intensities (Figure 3f), demonstrating reduced electron–hole recombination via enhanced charge transfer after hybridization. Additionally, the electrochemical impedance spectroscopy (EIS) data in Figure 3g revealed smaller semicircle diameters for ZM nanocomposites, confirming the hybridization‐induced promotion of charge transport kinetics [64]. The sulfur‐doping‐assisted enhancements of interfacial electron injection into ZIS, visible light absorptivity, and charge transport collectively contributed to superior photocatalytic activity of the ZM nanocomposites.

To further explore the benefits of hybridization with MAOS on the CO_2_RR activity of ZIS, DFT calculations were performed on the ZIS and MAOS/ZIS models based on experimental results (Figure S27). Firstly, the adsorption energies of ZIS and ZM25 for CO_2_ were respectively calculated to be −0.03 eV and −0.1 eV, supporting the promoted adsorption of CO_2_ after hybridization. Additionally, the Gibbs free energy (Δ*G* = Δ*E* + ΔZPE − *T*Δ*S*, Δ*E*: electronic energy, ΔZPE: zero‐point energy, Δ*S*: entropy) of ZIS and ZM25 for the CO_2_ reduction pathway was computed. Initially, Δ*E*, ΔZPE, and Δ*S* corrections of the adsorbed intermediates were derived from the vibrational frequency calculations, as listed in Tables S12−S14. Afterwards, the Gibbs free energy profiles of the CO_2_RRs occurring over the ZIS and ZM25 were systematically compared (Figure 3h). The free energy profiles indicated that ZM25 exhibited the lowest adsorption energy for CO_2_, attributing its high activity to the initial adsorption and activation of CO_2_. Notably, the positive CO_2_ adsorption energy predicted for ZM25 by ground‐state DFT originates from the lack of photoexcitation effects in the calculation. In fact, such moderately weak adsorption can be advantageous because it helps balance the initial activation and subsequent conversion of CO_2_, ultimately lowering the energy barrier associated with the rate‐determining step [65, 66]. Subsequently, during the proton‐coupled electron transfer (PCET) step for *COOH formation in which protons were supplied by H_2_O in the aqueous mixture, ZM25 exhibited a more favorable free energy change (−0.42 eV) compared with ZIS (−0.30 eV), indicating an enhanced thermodynamic driving force for intermediate stabilization and CO_2_ activation. This more exergonic *COOH formation suggests that ZM25 could more effectively facilitate the initial PCET process, thereby promoting the generation of key reaction intermediates. Moreover, the water dissociation energy on ZM25 was significantly reduced to 0.15 eV, in contrast to that on ZIS (1.13 eV, Table S15), indicating that proton supply from water was more kinetically accessible on ZM25. Particularly, in the critical step involving the conversion of *COOH to *CO, the ZM25 heterojunction crossed the lowest energy barrier of 0.32 eV, significantly lower than that of ZIS (0.76 eV). These theoretical calculations confirm that the hybridization with MAOS optimized the reaction pathways for CO_2_RR.

### 2.5 Underlying Mechanism for the Improved Photocatalytic CO_2_RR Activity

The mechanism underlying the improved CO_2_RR activity of the ZM nanocomposite was systematically investigated using combined in situ spectroscopic analyses before and after visible‐light irradiation. As shown in Figure 4a, in situ Zn K‐edge FT‐EXAFS analysis revealed a significant reduction in the intensity of the first FT peak associated with the Zn–S bonding pair in ZM25 (i.e., ZM25(on)) after visible‐light irradiation, which was partially restored after the removal of visible light (i.e., ZM25(off)). Pristine ZIS displayed similar but weaker photoinduced changes (Figure 4a). This trend was corroborated by the EXAFS fitting analyses, revealing a decrease in the coordination number of the Zn–S bonding pair after illumination, followed by an increase after light removal (Figure 4b and Table S16). These photoinduced changes in the coordination number were stronger for ZM25 than for ZIS, emphasizing that the structural frustration of local atomic arrangements around the Zn ions caused by photoinduced reduction of metal center and/or interactions with adsorbed reactant molecules occurred more actively after hybridization [67]. Similar changes in the FT peak intensity and coordination number were also discernible in the in situ In K‐edge FT‐EXAFS data (Figure 4c). However, the photoinduced spectral changes appeared weaker in the In K‐edge spectra than those in the Zn K‐edge spectra (Figure 4b and Table S17). This finding revealed that the Zn ions acted as the main reaction sites for the photocatalytic CO_2_RR.

**FIGURE 4:**
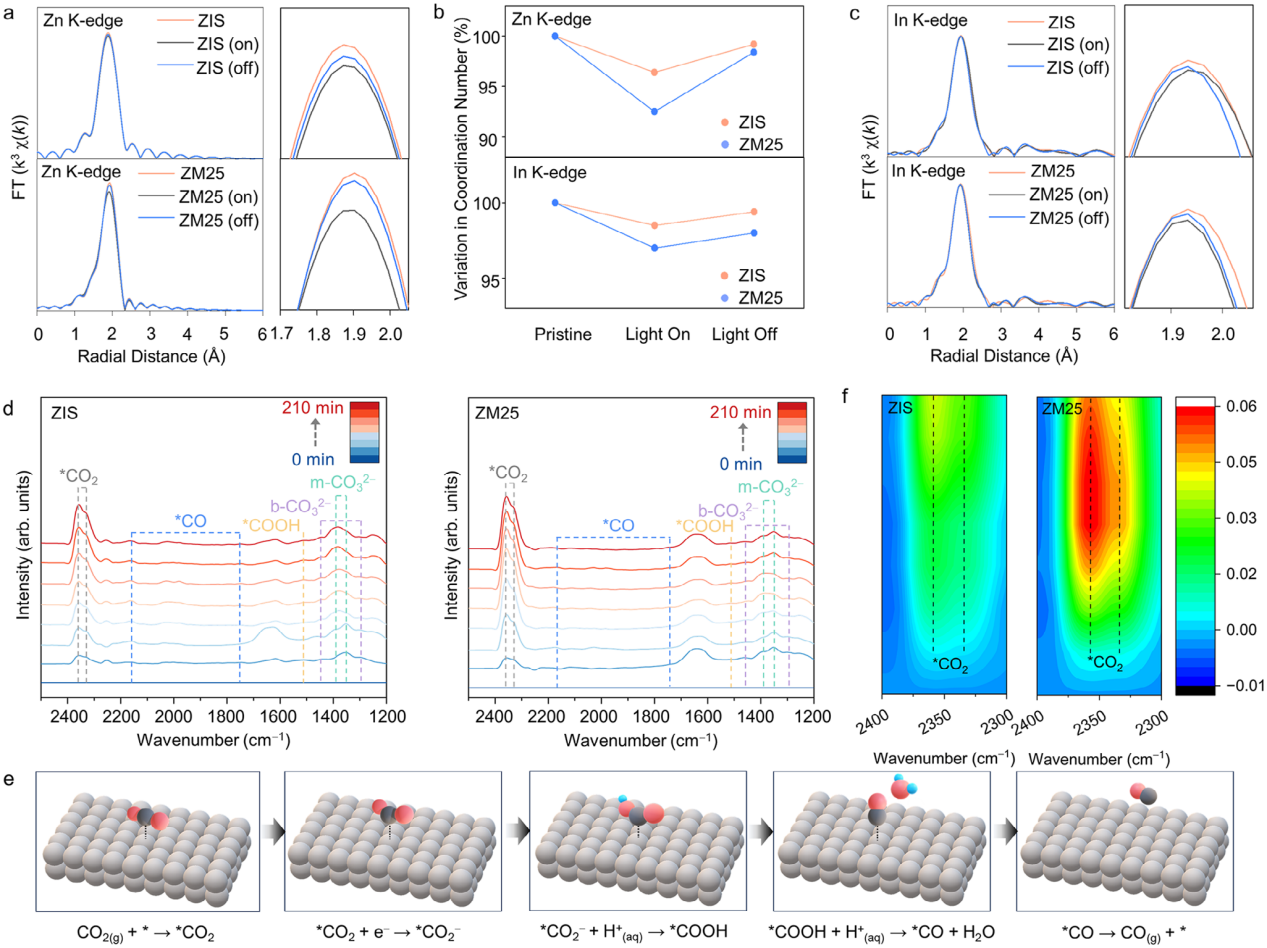
a) Zn K‐edge FT‐EXAFS data, b) plot of coordination number variation, and c) In K‐edge FT‐EXAFS data for ZIS and ZM25. d) In situ ATR‐FTIR spectra and f) intensity difference plots of ZIS and ZM25 (CO_2_ purging during 0−30 min, and visible irradiation during 30−210 min). e) Scheme for CO_2_RR operation mechanism of ZM25 nanocomposite.

Additionally, the reaction pathway was probed with in situ attenuated total reflectance‐Fourier transform infrared spectroscopy (ATR‐FTIR). As shown in Figure 4d, after introducing CO_2_ gas for 30 min, both ZIS and ZM25 displayed weak characteristic vibration peaks corresponding to adsorbed carbon dioxide in the forms of *CO_2_ at 2296–2362 cm^−1^, bidentate CO_3_^2−^ (b‐CO_3_^2−^) at 1316–1670 cm^−1^, and monodentate CO_3_^2−^ (m‐CO_3_^2−^) at 1292–1540 cm^−1^. Furthermore, the reaction intermediates, namely, *COOH and *CO were detected at 1511–1648 and 1715–2160 cm^−1^, respectively. After visible light irradiation, the intensities of these vibration peaks increased steadily for both ZIS and ZM25, revealing that CO_2_ activation and conversion were mainly induced by the energy of the incident photons. Based on these results, the reaction mechanism for the photocatalytic CO_2_ conversion can be proposed as follows: (1) CO_2(g)_ + * → *CO_2_, (2) *CO_2_ + e^−^ → *CO_2_^−^, (3) *CO_2_^−^ + H^+^_(aq)_ → *COOH, (4) *COOH + H^+^_(aq)_ → *CO + H_2_O, and (5) *CO → CO_(g)_ + * (Figure 4e). As depicted in Figure 4f, ZM25 displayed significantly stronger peaks corresponding to *CO_2_. This result offers compelling evidence for the hybridization‐induced promotion of CO_2_ adsorption, activation, and conversion. The enhanced adsorptivity of ZM25 toward Lewis acidic CO_2_ molecules could be ascribed to the interfacial charge transfer from MAOS to ZIS, resulting in an increase in the electron density and Lewis basicity of the hybridized ZIS. Additionally, the migration of adsorbed CO_2_ molecules from MAOS to the neighboring ZIS contributed to an enhanced CO_2_RR activity at ZIS domains.

The hybridization‐induced enhancement of interfacial charge transfer was further evidenced by in situ electron paramagnetic resonance (EPR) investigation using TEMPO as a radical scavenger (Figure S28a); ZM25 displayed lower EPR peak intensities in the TEMPO–e^−^ spectra than ZIS, highlighting the hybridization‐assisted enhancement of photoexcited electron transfer to TEMPO. This enhanced electron transfer could contribute significantly to the improved photocatalytic activity of ZM25 toward syngas production. Conversely, no significant difference in the EPR intensity was observed in the TEMPO–h^+^ spectra, demonstrating a negligible improvement in the hole transfer efficiency after hybridization (Figure S28b).

## 3 Conclusion

In this study, a surface bond competition approach was developed to explore efficient CO_2_ adsorbents and photocatalysts for syngas production through the sulfur‐doping‐induced control of the surface basicity of LDO nanosheets and subsequent hybridization with ZIS. DFT calculations and benzoic acid titrations clearly demonstrated that the incorporation of metal–sulfur bonds via CS_2_ treatment effectively increased the surface basicity of the MAOS nanosheets, which was achieved through bond competition with the strongly covalent Mg–S bonds. The resulting enhanced surface basicity of MAOS significantly increased its CO_2_ adsorptivity. Additionally, the sulfur‐doping of MAOS increased its Lewis softness, thus amplifying interfacial chemical interactions with metal‐sulfide photocatalysts. Hybridization between sulfur‐doped MAOS and HER‐active ZIS provided a powerful strategy for the development of efficient syngas production photocatalysts via the enhancement of CO_2_ adsorptivity and CO_2_RR activity. DFT calculations revealed that hybridization with MAOS nanosheets was effective in lowering both the adsorption energy of CO_2_ and the energy barrier for the conversion of *COOH to *CO. In situ EXAFS and in situ ATR‐FTIR spectroscopic analyses of ZM25 demonstrated photoinduced reversible local structural frustration and enhanced CO_2_ adsorption, highlighting the hybridization‐assisted enhancement of Lewis acid−base interactions between CO_2_ and ZIS. The hybridization‐induced promotion of electron injection into adsorbed CO_2_ molecules was further corroborated by in situ EPR spectroscopy. As summarized in Figure 5, the improved syngas photoproduction efficiency of ZM25 can be ascribed to the following factors. First, effective interfacial charge transfer from sulfur‐doped MAOS nanosheets enhanced the Lewis basicity of hybridized ZIS, promoting interactions with Lewis acidic CO_2_ molecules and accelerating the CO_2_RR. Second, the sulfur‐doping‐reinforced electronic correlation between the ZIS and MAOS nanosheets effectively promoted the spatial separation of photogenerated electrons and holes, preventing charge recombination. Third, the incorporation of the MAOS nanosheets enhanced the absorption of visible light. Fourth, the hybridization‐induced improvement in the charge transport properties facilitated the migration of charge carriers to the surface reaction sites. Finally, the hybridization with MAOS optimized the reaction pathways for CO_2_RR by lowering the energy barrier. Considering that the sulfur‐doping‐induced enhancement of CO_2_ adsorptivity appeared more prominent than the improvements of the optical and transport properties, the intensified Lewis acid−base interactions between adsorbed CO_2_ and hybridized ZIS was considered as the main contributor to the enhanced CO_2_RR photocatalytic activity of ZM nanocomposite. Taking into account the possibility of a phosphorus‐doping strategy in modulating the surface basicity and interfacial interactions in nanocomposites, this work effectively widens the spectrum of high‐performance CO_2_ adsorbents and photocatalysts toward CO_2_RR and syngas production via heteroatom doping (e.g., P, S) of various metal compounds (i.e., metal hydroxides and metal oxides) and subsequent hybridization.

**FIGURE 5:**
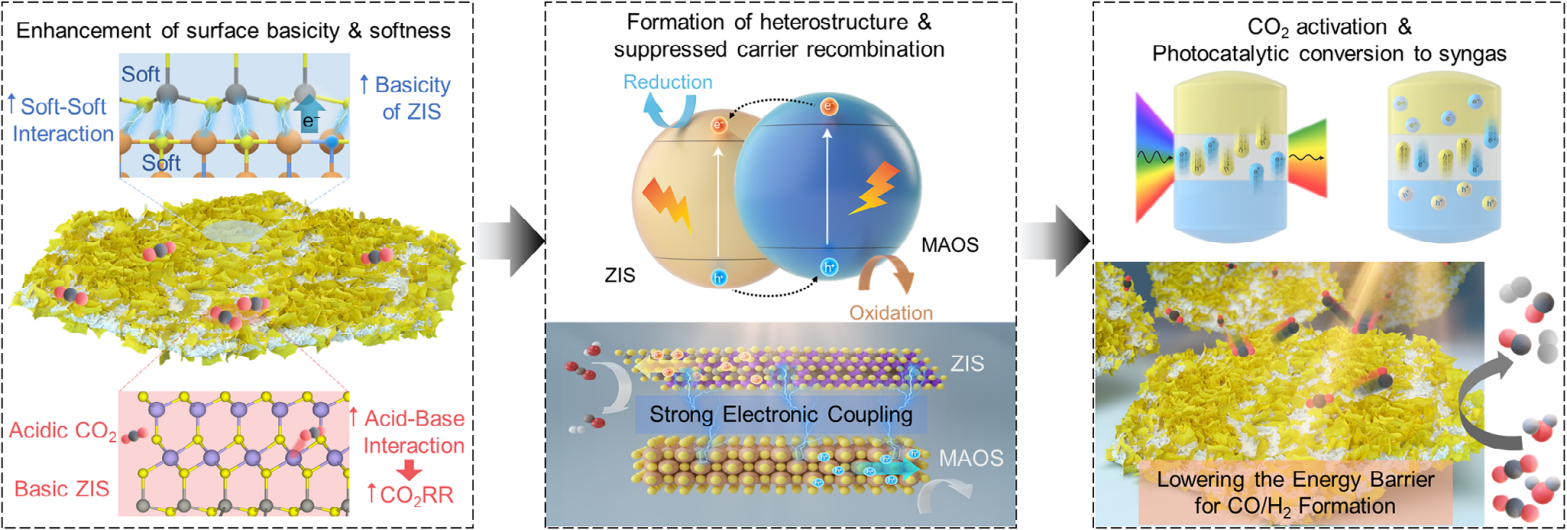
Schematic illustration of contributing factors to the enhanced photocatalytic conversion of CO_2_ to syngas in the ZM nanocomposite.

The authors have cited additional references within the Supporting Information [68, 69, 70, 71, 72, 73, 74, 75, 76, 77, 78, 79, 80, 81, 82].

## Author Contributions

**Taehoon Kim**: methodology, formal analysis, and investigation. **Nam Hee Kwon**: methodology, formal analysis, and investigation. **Xiaoyan Jin**: writing – review and editing, methodology, formal analysis, supervision, and investigation. **Wenjing Dong**: methodology, formal analysis, investigation, writing – original draft, and data curation. **In Young Kim**: formal analysis and methodology. **Ajayan Vinu**: writing – review and editing, methodology, supervision, formal analysis, and investigation. **Dong Wook Lee**: methodology, investigation, writing – original draft, formal analysis, and data curation. **Yufei Zhao**: conceptualization, investigation, writing – review and editing, methodology, formal analysis, and supervision. **Yun Kyung Jo**: formal analysis and methodology. **Seong‐Ju Hwang**: conceptualization, investigation, funding acquisition, writing – original draft, writing – review and editing, methodology, formal analysis, and supervision, data curation.

## Conflicts of Interest

The authors declare no conflicts of interest.

## Supporting information




**Supporting file**: advs76890‐sup‐0001‐SuppMat.docx

## Data Availability

The data that support the findings of this study are available from the corresponding author upon reasonable request.

## References

[1] Z. Liu , Z. Deng , S. J. Davis , and P. Ciais , “Global Carbon Emissions in 2023,” Nature Reviews Earth & Environment 5, no. 4 (2024): 253–254, 10.1038/s43017-024-00532-2. PMC10010646 37065615

[2] J. A. Rudd , “An Industrial Take on Developing and Deploying Carbon Capture at Scale,” Nature Reviews Chemistry 8, (2024): 1–2, 10.1038/s41570-023-00560-4. 38036685

[3] M. I. Vousdoukas , P. Athanasiou , A. Giardino , et al., “Small Island Developing States under Threat by Rising Seas Even in a 1.5°C Warming World,” Nature Sustainability 6, (1522): 1552–1564, 10.1038/s41893-023-01230-5.

[4] W.‐P. Chan , J. Lenoir , G.‐S. Mai , H.‐C. Kuo , I.‐C. Chen , and S.‐F. Shen , “Climate Velocities and Species Tracking in Global Mountain Regions,” Nature 629, no. 8010 (2024): 114–120, 10.1038/s41586-024-07264-9. PMC11062926 38538797

[5] K. C. Poon , W. Y. Wan , H. Su , and H. Sata , “A Review on Recent Advances in the Electrochemical Reduction of CO_2_ to CO with Nano‐Electrocatalysts,” RSC Advances 12, no. 35 (2022): 22703–22721, 10.1039/D2RA03341K. PMC9376860 36105973

[6] K. Wang , Y. Hu , X. Liu , J. Li , and B. Liu , “Visible‐Light‐Driven CO_2_ Photoreduction over Atomically Strained Indium Sites in Ambient Air,” Nature Communications 16, no. 1 (2025): 2094, 10.1038/s41467-025-57140-x. PMC11873254 40025011

[7] Q. Zhong , Y. Sun , S.‐G. Yang , et al., “Anion Vacancies Triggered Spin Polarization Enables Efficient Piezocatalysis for Water Cleanup,” Angewandte Chemie International Edition 64, no. 32 (2025): 202507265, 10.1002/anie.202507265. 40491318

[8] J. Lee , T. Kim , D. H. Sun , X. Jin , and S.‐J. Hwang , “Recent Advances in Two‐Dimensional Metal Pnictogenide Nanosheets and Their Nanohybrids with Diverse Energy Applications,” EnergyChem 7, no. 1 (2025): 100139, 10.1016/j.enchem.2024.100139.

[9] X. Wang , X. Sang , C.‐L. Dong , et al., “Proton Capture Strategy for Enhancing Electrochemical CO_2_ Reduction on Atomically Dispersed Metal–Nitrogen Active Sites,” Angewandte Chemie International Edition 60, (2021): 11959–11965, 10.1002/anie.202100011. 33599063

[10] Y.‐J. Wang , X. Cheng , N.‐N. Ma , et al., “In Situ Growth of Metal‐Organic Layer on Polyoxometalate‐Etching Cu_2_O to Boost CO_2_ Reduction with High Stability,” Angewandte Chemie International Edition 64, no. 13 (2025): 202423204, 10.1002/anie.202423204. 39777831

[11] W. Ma , J. Sun , S. Yao , et al., “Synergistic Interplay of Dual‐Active‐Sites on Metallic Ni‐MOFs Loaded with Pt for Thermal‐Photocatalytic Conversion of Atmospheric CO_2_ under Infrared Light Irradiation,” Angewandte Chemie International Edition 62, (2023): 202313784, 10.1002/anie.202313784. 37819255

[12] Y. K. Jo , M. Kim , X. Jin , et al., “Hybridization of a Metal–Organic Framework with a Two‐Dimensional Metal Oxide Nanosheet: Optimization of Functionality and Stability,” Chemistry of Materials 29, no. 3 (2017): 1028–1035, 10.1021/acs.chemmater.6b03788.

[13] R. Vismara , S. Terruzzi , A. Maspero , et al., “CO_2_ Adsorption in a Robust Iron(III) Pyrazolate‐Based MOF: Molecular‐Level Details and Frameworks Dynamics from Powder X‐Ray Diffraction Adsorption Isotherms,” Advanced Materials 36, no. 12 (2024): 2209907, 10.1002/adma.202209907. 36735860

[14] R. A. Maia , B. Louis , W. Gao , and Q. Wang , “CO_2_ Adsorption Mechanisms on MOFs: A Case Study of Open Metal Sites, Ultra‐Microporosity and Flexible Framework,” Reaction Chemistry & Engineering 6, (2021): 1118, 10.1039/D1RE00090J.

[15] Y. Shen , F. Liu , X. Wang , et al., “A Pore Matching Amine‐Functionalized Strategy for Efficient CO_2_ Physisorption with Low Energy Penalty,” Chemical Engineering Journal 432, (2022): 134403, 10.1016/j.cej.2021.134403.

[16] S. Yang , W. Zhang , G. Pan , et al., “Photocatalytic Co‐Reduction of N_2_ and CO_2_ with CeO_2_ Catalyst for Urea Synthesis,” Angewandte Chemie International Edition 62, (2023): 202312076, 10.1002/anie.202312076. 37667537

[17] Y. H. Park , Y. Sun , D. W. Lee , I. Y. Kim , X. Jin , and S.‐J. Hwang , “Recent Trends in Combinative Defect Engineering and Hybridization Approach to Develop Efficient Energy‐Functional Layered Double Hydroxide‐Based Materials,” Nano Research Energy 4, no. 2 (2025): 9120163, 10.26599/NRE.2025.9120163.

[18] T. Kim , Y. H. Park , J. Lee , A. Vinu , and S.‐J. Hwang , “Phase‐Tunable Exsolution Approaches to Energy‐Functional Nanostructured Metal‐Based Hybrid Materials: Synthesis, Application, and Perspective,” EnergyChem 8, no. 4 (2026): 100201, 10.1016/j.enchem.2026.100201.

[19] L. Jin , X. Zhou , F. Wang , et al., “Insights into Memory Effect Mechanisms of Layered Double Hydroxides with Solid‐State NMR Spectroscopy,” Nature Communications 13, no. 1 (2022): 6093, 10.1038/s41467-022-33912-7. PMC9568524 36241633

[20] C. Li , R. Guo , Z. Zhang , et al., “Constructing CoAl‐LDO/MoO_3−x_ S‐Scheme Heterojunctions for Enhanced Photocatalytic CO_2_ Reduction,” Journal of Colloid and Interface Science 650, (2023): 983–993, 10.1016/j.jcis.2023.07.068. 37453322

[21] R. Chong , C. Su , Y. Du , et al., “Insights into the Role of MgAl Layered Double Oxides Interlayer in Pt/TiO_2_ toward Photocatalytic CO_2_ Reduction,” Journal of Catalysis 363, (2018): 92–101, 10.1016/j.jcat.2018.04.020.

[22] J. E. Huheey , E. A. Keiter , and R. L. Keiter , Inorganic Chemistry: Principles of Structure and Reactivity (Harpercollins College Publishers, 1993): 138–251.

[23] D. K. Chauhan , N. Sharma , and K. Kailasam , “A Critical Review on Emerging Photocatalysts for Syngas Generation via CO_2_ Reduction under Aqueous Media: A Sustainable Paradigm,” Materials Advances 3, no. 13 (2022): 5274–5298, 10.1039/d2ma00334a.

[24] W. Sun , H. Zhang , N. Gao , et al., “Morphology‐Engineered ZnIn_2_S_4_ Nanoflowers on GaN Photoanodes for Efficient Solar‐Driven CO_2_‐to‐Syngas Conversion,” Advanced Energy Materials 28, (2026): 202502345, 10.1002/adem.202502345.

[25] A. Yohannes and I. Gates , “A Century of Evolution: Progress and Milestones in Fischer‐Tropsch Synthesis,” Coordination Chemistry Reviews 547, (2026): 217096, 10.1016/j.ccr.2025.217096.

[26] S. Lögdberg , D. Tristantini , Ø. Borg , et al., “Hydrocarbon Production via Fischer–Tropsch Synthesis from H_2_‐Poor Syngas over Different Fe‐Co/γ‐Al_2_O_3_ Bimetallic Catalysts,” Applied Catalysis B: Environmental 89, (2009): 167–182, 10.1016/j.apcatb.2008.11.037.

[27] J. Jack , J. Lo , P.‐C. Maness , and Z. J. Ren , “Directing *Clostridium Ljungdahlii* Fermentation Products via Hydrogen to Carbon Monoxide Ratio in Syngas,” Biomass and Bioenergy 124, (2019): 95–101, 10.1016/j.biombioe.2019.03.011.

[28] M. Diender , A. J. M. Stams , and D. Z. Sousa , “Production of Medium‐Chain Fatty Acids and Higher Alcohols by a Synthetic Co‐Culture Grown on Carbon Monoxide or Syngas,” Biotechnology for Biofuels 9, no. 1 (2016): 82, 10.1186/s13068-016-0495-0. PMC4818930 27042211

[29] C. Peinado , D. Liuzzi , S. N. Sluijter , et al., “Review and Perspective: Next Generation DME Synthesis Technologies for the Energy Transition,” Chemical Engineering Journal 479, (2024): 147494, 10.1016/j.cej.2023.147494.

[30] H. Jahangiri , J. Bennett , P. Mahjoubi , K. Wilson , and S. Gu , “A Review of Advanced Catalyst Development for Fischer–Tropsch Synthesis of Hydrocarbons from Biomass Derived Syn‐Gas,” Catalysis Science & Technology 4, no. 8 (2014): 2210–2229, 10.1039/c4cy00327f.

[31] I. Y. Kim , J. Seo , S. M. Oh , S. B. Patil , and S.‐J. Hwang , “Situ Formation of Conductive Metal Sulfide Domain in Metal Oxide Matrix: an Efficient Way to Improve the Electrochemical Activity of Semiconducting Metal Oxide,” Advanced Functional Materials 25, (2015): 4948, 10.1002/adfm.201501478.

[32] V. K. Abdelkader‐Fernández , M. Domingo‐García , F. J. López‐Garzón , et al., “Expanding Graphene Properties by a Simple S‐Doping Methodology Based on Cold CS_2_ Plasma,” Carbon 144, (2019): 269, 10.1016/j.carbon.2018.12.045.

[33] D. Scarano , S. Bertarione , A. Zecchina , R. Soave , and G. Pacchioni , “Adsorption of CS_2_ on MgO Microcrystals: Formation of a S‐Doped MgO Surface,” Physical Chemistry Chemical Physics 4, no. 2 (2002): 366–374, 10.1039/B104566K.

[34] X. Zhao , F. Zhang , S. Xu , D. G. Evans , and X. Duan , “From Layered Double Hydroxides to ZnO‐Based Mixed Metal Oxides by Thermal Decomposition: Transformation Mechanism and UV‐Blocking Properties of the Product,” Chemistry of Materials 22, no. 13 (2010): 3933–3942, 10.1021/cm100383d.

[35] Y. Gao , Z. Zhang , J. Wu , et al., “Comprehensive Investigation of CO_2_ Adsorption on Mg–Al–CO_3_ LDH‐Derived Mixed Metal Oxides,” Journal of Materials Chemistry A 1, no. 41 (2013): 12782, 10.1039/C3TA13039H.

[36] L. Pauling , The Nature of the Chemical Bond (Cornell University Press, 1960).

[37] X. Lu , C. Zhao , A. Chen , et al., “Reducing Zn‐Ion Concentration Gradient by SO_4_ ^2−^‐Immobilized Interface Coating for Dendrite‐Free Zn Anode,” Chemical Engineering Journal 451, (2023): 138772, 10.1016/j.cej.2022.138772.

[38] P. Larkin , Infrared and Raman Spectroscopy (Elsevier, 2011), 117–133.

[39] D. R. Neuville , D. de Ligny , L. Cormier , et al., “The Crystal and Melt Structure of Spinel and Alumina at High Temperature: An In‐Situ XANES Study at the Al and Mg K‐Edge,” Geochimica et Cosmochimica Acta 73, no. 11 (2009): 3410–3422, 10.1016/j.gca.2009.02.033.

[40] T. Yoshida , T. Tanaka , H. Yoshida , T. Funabiki , S. Yoshida , and T. Murata , “Study of Dehydration of Magnesium Hydroxide,” The Journal of Physical Chemistry 99, no. 27 (1995): 10890–10896, 10.1021/j100027a033.

[41] S. M. Davis , Y. Zhou , M. Freeman , D. A. Fischer , G. M. Meitzner , and J. L. Gland , “Carbon K‐Edge X‐Ray Absorption Spectroscopy of Gas Oil Derived Coke Deposits in LZ‐210 Zeolite,” Journal of Catalysis 139, no. 1 (1993): 322–325, 10.1006/jcat.1993.1026.

[42] S. L. Gregg and K. S. W. Sing , Adsorption, Surface Area and Porosity (Academic Press, 1980).

[43] S.‐M. Wu , Y.‐T. Wang , S.‐T. Xiao , et al., “Design and Synthesis of TiO_2_/C Nanosheets with a Directional Cascade Carrier Transfer,” Chemical Science 13, no. 24 (2022): 7126–7131, 10.1039/D2SC01872A. PMC9214889 35799830

[44] R. Bardestani , G. S. Patience , and S. Kaliaguine , “Experimental Methods in Chemical Engineering: Specific Surface Area and Pore Size Distribution Measurements—BET, BJH, and DFT,” The Canadian Journal of Chemical Engineering 97, no. 11 (2019): 2781–2791, 10.1002/cjce.23632.

[45] M. S. Shafeeyan , W. M. A. W. Daud , A. Shamiri , and N. Aghamohammadi , “Adsorption Equilibrium of Carbon Dioxide on Ammonia‐Modified Activated Carbon,” Chemical Engineering Research and Design 104, (2015): 42–52, 10.1016/j.cherd.2015.07.018.

[46] M. Longuet , K. Vitse , A. Martin‐Mingot , B. Michelet , F. Guegan , and S. Thibaudeau , “Determination of the Hammett Acidity of HF/Base Reagents,” Journal of the American Chemical Society 146, no. 17 (2024): 12167–12173, 10.1021/jacs.4c02344. 38626381

[47] D. Cornu , H. Guesmi , G. Laugel , J.‐M. Krafft , and H. Lauron‐Pernot , “On the Relationship between the Basicity of a Surface and Its Ability to Catalyze Transesterification in Liquid and Gas Phases: the Case of MgO,” Physical Chemistry Chemical Physics 17, no. 21 (2015): 14168–14176, 10.1039/C5CP00217F. 25958788

[48] S. Tosoni , D. Spinnato , and G. Pacchioni , “DFT Study of CO_2_ Activation on Doped and Ultrathin MgO Films,” The Journal of Physical Chemistry C 119, no. 49 (2015): 27594–27602, 10.1021/acs.jpcc.5b10130.

[49] A. Haaland , “Covalent versus Dative Bonds to Main Group Metals, a Useful Distinction,” Angewandte Chemie International Edition in English 28, no. 8 (1989): 992–1007, 10.1002/anie.198909921.

[50] Q. Li , X. Li , M. Zheng , et al., “Spatial Coupling of Photocatalytic CO_2_ Reduction and Selective Oxidation on Covalent Triazine Framework/ZnIn_2_S_4_ Core–Shell Structures,” Advanced Functional Materials 35, no. 11 (2024): 2417279, 10.1002/adfm.202417279.

[51] X. Wang , X. Wang , J. Huang , S. Li , A. Meng , and Z. Li , “Interfacial Chemical Bond and Internal Electric Field Modulated Z‐Scheme S_v_‐ZnIn_2_S_4_/MoSe_2_ Photocatalyst for Efficient Hydrogen Evolution,” Nature Communications 12, no. 1 (2021): 4112, 10.1038/s41467-021-24511-z. PMC8257585 34226543

[52] H. Fan , M. Hu , Y. Duan , et al., “Hollow Core–shell Heterojunction TAPB‐COF@ZnIn_2_S_4_ as Highly Efficient Photocatalysts for Carbon Dioxide Reduction,” Chemical Science 16, no. 5 (2025): 2316–2324, 10.1039/D4SC07077A. PMC11701727 39776658

[53] Y. Sun , K. Lai , X. Shi , N. Li , Y. Gao , and L. Ge , “Regulating Metal Cation Cu Vacancies on ZnIn_2_S_4_/Cu_1.81_S to Achieve High Selectivity for the Photocatalytic Reduction of CO_2_ to CH_4_ ,” Applied Catalysis B: Environment and Energy 365, (2025): 124907, 10.1016/j.apcatb.2024.124907.

[54] V. Dřínek , P. Dytrych , R. Fajgar , et al., “A Robust and High Performance Copper Silicide Catalyst for Electrochemical CO_2_ Reduction,” Materials Advances 5, (2024): 2917–2925, 10.1039/d3ma00633f.

[55] C. Chen , Y. Pang , F. Zhang , J. Zhong , B. Zhang , and Z. Cheng , “Sharp Cu@Sn Nanocones on Cu Foam for Highly Selective and Efficient Electrochemical Reduction of CO_2_ to Formate,” Journal of Materials Chemistry A 6, no. 40 (2018): 19621–19630, 10.1039/c8ta06826g.

[56] X. Yi , S. Zhang , H. Shen , et al., “Atomic Sulfur Dissimilation Remolding ZnIn_2_S_4_ Nanosheets Surface to Enhance Built‐Internal Electric Field for Photocatalytic CO_2_ Conversion to Syngas,” Applied Catalysis B: Environmental 338, (2023): 123003, 10.1016/j.apcatb.2023.123003.

[57] S. Yuan , Y. Feng , S. Liang , et al., “Synergistic Effect of Conjugation Degree of Acceptors in D‐A Azine‐Based‐COF and Defects of ZnIn_2_S_4_ on Charge Separation of COF/ZnIn_2_S_4_ Heterojunctions for Enhanced Photocatalytic CO_2_ Reduction,” Materials Today Energy 48, (2025): 101804, 10.1016/j.mtener.2025.101804.

[58] G. Zhang , Z. Wang , T. He , J. Wu , J. Zhang , and J. Wu , “Rationally Design and In‐Situ Fabrication of Ultrasmall Pomegranate‐Like CdIn_2_S_4_/ZnIn_2_S_4_ Z‐Scheme Heterojunction with Abundant Vacancies for Improving CO_2_ Reduction and Water Splitting,” Chemical Engineering Journal 442, (2022): 136309, 10.1016/j.cej.2022.136309.

[59] K.‐L. Xie , Y.‐Q. Liao , J.‐J. Hu , K.‐Q. Lu , and H.‐R. Wen , “Rationally Designed S‐Scheme CeO_2_/G‐C_3_N_4_ Heterojunction for Promoting Visible Light Driven CO_2_ Photoreduction into Syngas,” ChemSusChem 17, no. 23 (2024): 202400969, 10.1002/cssc.202400969. 38874368

[60] Y. Liu , A. Deng , Y. Yin , et al., “Modulation of Catalyst Microenvironments in ZnIn_2_S_4_/G‐C_3_N_4_ S‐Scheme Heterojunction for Ratio‐Tunable Syngas Production from CO_2_ Photoreduction,” Applied Catalysis B: Environment and Energy 362, (2025): 124724, 10.1016/j.apcatb.2024.124724.

[61] H. Chen , Y. Xiong , J. Li , et al., “Epitaxially Grown Silicon‐Based Single‐Atom Catalyst for Visible‐Light‐Driven Syngas Production,” Nature Communications 14, no. 1 (2023): 1719, 10.1038/s41467-023-37401-3. PMC10050177 36977716

[62] M. Li , C. Li , Y. Shi , et al., “Embedding Metallosalen Active Sites in Zr‐MOF for Enhanced Selective Syngas Production from CO_2_ Photoreduction,” Angewandte Chemie International Edition 64, no. 39 (2025): 202510810, 10.1002/anie.202510810. 40761025

[63] K. Gong , F. Wang , K. Zhang , et al., “One‐Step Molten Salt Inducing Copper(I) and Sulfur Vacancies Decoration in In_2_S_3_ Nanocrystals to Regulate Photocatalytic CO_2_ Reduction to Syngas,” Advanced Sustainable Systems 10, no. 3 (2026): 00005, 10.1002/adsu.202600005.

[64] N. H. Kwon , J. Park , X. Jin , S.‐J. Kim , H. Kim , and S.‐J. Hwang , “Defect‐Regulated Two‐Dimensional Superlattice of Holey g‐C_3_N_4_–TiO_2_ Nanohybrids: Contrasting Influence of Vacancy Content on Hybridization Impact and Photocatalyst Performance,” ACS Nano 17, no. 23 (2023): 23732–23745, 10.1021/acsnano.3c07566. 38039389

[65] S. Wei , Y. Xu , T. Song , et al., “Steering the Absorption Configuration of Intermediates over Pd‐Based Electrocatalysts toward Efficient and Stable CO_2_ Reduction,” Journal of the American Chemical Society 147, no. 5 (2025): 4219–4229, 10.1021/jacs.4c14253. PMC11803748 39854612

[66] Y. Yang , J. Jiang , Y.‐N. Feng , et al., “Manipulating d‐Band Center and d–p Orbital Coupling to Mitigate Adsorption–desorption Trade‐off for Efficient CO_2_‐to‐CO Photoreduction over MOF Nanofibers,” Applied Catalysis B: Environment and Energy 379, (2025): 125643, 10.1016/j.apcatb.2025.125643.

[67] H. Zhang , S. Zuo , M. Qiu , et al., “Direct Probing of Atomically Dispersed Ru Species over Multi‐Edged TiO_2_ for Highly Efficient Photocatalytic Hydrogen Evolution,” Science Advances 6, (2020): abb9823, 10.1126/sciadv.abb9823. PMC7531879 32967834

[68] L. Li , R. Ma , T. Ebina , N. Iyi , and T. Sasaki , “Positively Charged Nanosheets Derived via Total Delamination of Layered Double Hydroxides,” Chemistry of Materials 17, no. 17 (2005): 4386–4391, 10.1021/cm0510460.

[69] G. Kresse and J. Furthmüller , “Efficiency of Ab‐Initio Total Energy Calculations for Metals and Semiconductors Using a Plane‐Wave Basis Set,” Computational Materials Science 6, no. 1 (1996): 15–50, 10.1016/0927-0256(96)00008-0. 9984901

[70] G. Kresse and J. Furthmüller , “Efficient Iterative Schemes for Ab Initio Total‐Energy Calculations Using a Plane‐Wave Basis Set,” Physical Review B 54, no. 16 (1996): 11169–11186, 10.1103/PhysRevB.54.11169. 9984901

[71] J. P. Perdew , K. Burke , and M. Ernzerhof , “Generalized Gradient Approximation Made Simple,” Physical Review Letters 77, no. 18 (1996): 3865–3868, 10.1103/PhysRevLett.77.3865. 10062328

[72] P. Geysermans , F. Finocchi , J. Goniakowski , R. Hacquart , and J. Jupille , “Combination of (100), (110) and (111) Facets in MgO Crystals Shapes from Dry to Wet Environment,” Physical Chemistry Chemical Physics 11, no. 13 (2009): 2228–2233, 10.1039/b812376d. 19305895

[73] M. Sassi and K. M. Rosso , “First Principles Simulations of MgO(100) Surface Hydration at Ambient Conditions,” Physical Chemistry Chemical Physics 26, no. 3 (2024): 2269–2276, 10.1039/d3cp04848a. 38165646

[74] T. Kameda , S. Nagano , S. Kumagai , Y. Saito , and T. Yoshioka , “Enrichment of Carbon Dioxide Using Mg–Al Layered Double Hydroxides,” Chemical Engineering Research and Design 194, (2023): 318–324, 10.1016/j.cherd.2023.04.065.

[75] D. W. J. Leung , K. R. Laney , P. Kenyon , et al., “Optimizing the Acid–Base Ratio of Mg–Al Layered Double Oxides to Enhance CO_2_ Capture Performance: the Critical Role of Calcination Conditions,” Dalton Transactions 53, (2024): 6200–6206, 10.1039/D4DT00270A. 38482861

[76] I. Maseeh , F. Anwar , S. Aroob , et al., “Multifunctional MgAl LDH/Zn‐MOF S‐Scheme Heterojunction: Efficient Hydrogen Production, Methyl Red Removal, and CO_2_ Adsorption,” Materials Advances 5, no. 12 (2024): 5080–5095, 10.1039/D4MA00038B.

[77] A. C. Faria , R. Trujillano , V. Rives , C. V. Miguel , A. E. Rodrigues , and L. M. Madeira , “Alkali Metal (Na, Cs and K) Promoted Hydrotalcites for High Temperature CO_2_ Capture from Flue Gas in Cyclic Adsorption Processes,” Chemical Engineering Journal 427, (2022): 131502, 10.1016/j.cej.2021.131502.

[78] L. Sun , Y. Yang , H. Ni , et al., “Enhancement of CO_2_ Adsorption Performance on Hydrotalcites Impregnated with Alkali Metal Nitrate Salts and Carbonate Salts,” Industrial & Engineering Chemistry Research 59, (2020): 6043–6052, 10.1021/acs.iecr.9b05700.

[79] A. C. Faria , R. Trujillano , V. Rives , C. V. Miguel , A. E. Rodrigues , and L. M. Madeira , “Cyclic Operation of CO_2_ Capture and Conversion into Methane on Ni‐Hydrotalcite Based Dual Function Materials (DFMs),” Journal of CO2 Utilization 72, (2023): 102476, 10.1016/j.jcou.2023.102476.

[80] D. Chaillot , V. Folliard , J. Miehé‐Brendlé , A. Auroux , L. Dzene , and S. Bennici , “Basic Properties of MgAl‐Mixed Oxides in CO_2_ Adsorption at High Temperature,” Materials 16, (2023): 5698, 10.3390/ma16165698. PMC10456579 37629987

[81] L. Santamaría , S. A. Korili , and A. Gil , “Metal‐Al Layered Double Hydroxides Synthesized from Aluminum Slags as Efficient CO_2_ Adsorbents at Pre‐ and Post‐Combustion Temperature,” Journal of Environmental Chemical Engineering 11, no. 5 (2023): 110936, 10.1016/j.jece.2023.110936.

[82] X. Ju , X. Feng , X. Duan , et al., “Interfacial Engineering of MXene‐Induced Nanoflower‐Like LDH Heterostructures for Enhanced CO_2_ Capture,” Separation and Purification Technology 385 (2026): 136443, 10.1016/j.seppur.2025.136443.

