# Supplementary material for: Bond‐Competition‐Driven Enhancement of Surface Basicity and Interfacial Interaction to Boost Photocatalytic Syngas Production
Source: Adv Sci (Weinh). 2026 Aug 3:e76890. Online ahead of print. doi: 10.1002/advs.76890 (PMC13430942; doi:10.1002/advs.76890)
Supplement: Supplementary file 1 — Supporting file: advs76890‐sup‐0001‐SuppMat.docx [file ADVS-9999-e76890-s001.docx]

Supporting Information

Bond-Competition-Driven Enhancement of Surface Basicity and Interfacial Interaction to Boost Photocatalytic Syngas Production

Dong Wook Lee,^[a,+]^ Wenjing Dong,^[b,+]^ Nam Hee Kwon,^[a]^ Taehoon Kim,^[a]^ In Young Kim,^[c]^ Yun Kyung Jo,^[c]^ Xiaoyan Jin,*^[d]^ Ajayan Vinu,*^[e]^ Yufei Zhao,*^[b]^ and Seong-Ju Hwang*^[a,f]^

[a] D. W. Lee, Dr. N. H. Kwon, T. Kim, Prof. S.-J. Hwang
Department of Materials Science and Engineering,
College of Engineering, Yonsei University
Seoul 03722, Republic of Korea
E-mail: hwangsju@yonsei.ac.kr

[b] W. Dong, Prof. Y. Zhao
State Key Laboratory of Chemical Resource Engineering,
Beijing University of Chemical Technology
Beijing, 100029, P. R. China
E-mail: zhaoyufei@buct.edu.cn

[c] Prof. I. Y. Kim, Dr. Y. K. Jo
Department of Chemistry and Nanoscience,
Ewha Womans University
Seoul 03760, Republic of Korea

[e] Prof. X. Jin
Department of Applied Chemistry,
University of Seoul
Seoul 02504, Republic of Korea
E-mail: xjin@uos.ac.kr

[f] Prof. A. Vinu
Global Innovative Center for Advanced Nanomaterials,
The University of Newcastle
Callaghan, NSW 2308, Australia
E-mail: Ajayan.Vinu@newcastle.edu.au

[f] Prof. S.-J. Hwang
Department of Battery Engineering,
Yonsei University
Seoul 03722, Republic of Korea

[+] These authors contributed equally to this work.

Experimental Procedures

**Sample preparation:**

The Mg–Al-layered double hydroxide (Mg–Al-LDH) precursor was prepared via a hydrothermal reaction using a precursor aqueous solution consisting of Mg(NO_3_)_2_⋅6H_2_O (0.02 M), Al(NO_3_)_3_⋅9H_2_O (0.01 M), and hexamethylenetetramine (0.026 M) at 140 °C for 24 h.^[68]^ The resulting product was subjected to centrifugation, washed thoroughly with deionized water to remove excess ions, and dried in oven at 50 °C for 12 h. The MgO/MgAl_2_O_4_S_x_ (MAOS) nanosheets were synthesized by reacting the precursor Mg–Al-LDH nanosheets at 450 °C for 2 h under a flow of CS_2_. The CS_2_ flow was generated by bubbling Ar carrier gas into ≥ 99% CS_2_ in a reaction vessel of 10 mL.^[31]^ The flow rate of the Ar carrier gas was set at 30 mL min^−1^. As a precursor for hybridization with the MAOS nanosheets, the ZnIn_2_S_4_ (ZIS) nanoplates were synthesized using a crystal growth method. Typically, ZnCl_2_, In(CH_3_COO)_3_, and thioacetamide were dissolved in a stoichiometric ratio, and the pH was adjusted to 2.5 using 1.0 M HCl solution. After allowing the reaction to proceed for 2 h, the resulting yellow precipitate was thoroughly washed with distilled water and ethanol, and dried in a vacuum oven at 60 °C for 4 h. Hybridization between ZIS and MAOS was conducted via the ball milling of a physical mixture of ZIS and MAOS nanosheets in ZIS/MAOS weight ratios of 10, 25, and 50 wt%. Ethanol was added to the ball-milling mixture as a coolant.

**Sample characterization:**

The crystal structures of the MAOS nanosheets and ZIS–MAOS (ZM) nanocomposites were determined using powder X-ray diffraction (XRD) (Rigaku D/Max-2000/PC) with a Ni-filtered Cu Ka irradiation source (λ = 1.5418 Å) at 25 °C. The crystallite morphologies of the samples were examined by field emission-scanning electron microscopy (FE-SEM, JEOL JSM-7610F) and transmission electron microscopy (TEM, JEOL JEM-ARM200F) at an accelerating voltage of 200 kV. The chemical compositions of the nanocomposites were estimated using inductively coupled plasma optical emission spectroscopy (ICP–OES, Agilent 5110) and energy dispersive spectroscopy (EDS) (JEOL JSM-7610F FE-SEM equipped with an EDS instrument). The micro-Raman spectra were recorded using a JY LabRam HR spectrometer, in which an Ar laser (λ = 785 nm) was used as the excitation source. X-ray photoelectron spectroscopy (XPS) was performed using a K-alpha spectrometer (Thermo VG, UK, Al Kα) equipped with a dual X-ray source to minimize the charging effect. The XPS data were obtained from a thin layer of the sample loaded on highly conductive copper foil to suppress charge accumulation, and were referenced to the adventitious C 1s peak at 285 eV. The Mg K-edge X-ray absorption near edge structure (XANES) experiments were carried out at the extended X-ray absorption fine structure (EXAFS) facility installed at the soft X-ray beamline at the Australian Synchrotron. The beamline was equipped with a hemispherical electron analyzer and a microchannel plate detector to simultaneously measure the total and partial electron yields. The raw XANES data were double-normalized to the photoelectron current of the photon beam measured on an Au grid. All spectra were calibrated using Mg foil as a reference. The surface areas and pore structures of the nanocomposites were examined by measuring their N_2_ adsorption–desorption isotherms at −196 °C (Micromeritics ASAP 2020). The CO_2_ adsorption experiments were performed at 0 and 300 °C using the same instrument. Prior to conducting the adsorption measurements, all materials were degassed at 150 °C for 5 h under vacuum. The surface basicity of each material was determined using the benzoic acid titration method, in which the material (10 mg) was suspended in phenolphthalein indicator solution (3 mL, 20 mg mL^−1^) prior to titration with a 0.01 M solution of benzoic acid in toluene. The hybridization-induced evolution of the optical properties was characterized using diffuse reflectance UV–vis (JASCO V-760) and photoluminescence (PL, Perkin Elmer FL 8500) spectroscopy. The Zn K-edge and In K-edge XANES/EXAFS spectra were measured at beamlines 8C and 10C, respectively, of the Pohang Accelerator Laboratory (PAL, Pohang, Republic of Korea). The in situ EXAFS data were obtained by home-made in situ cell. All XANES/EXAFS spectra were energy-calibrated by simultaneously measuring the reference spectra of Zn and In metal foils.

**DFT calculation and benzoic acid titration method:**

Plane-wave density functional theory (DFT) calculations were used to model MgO with/without partial doping by one S atom using the CASTEP module in Materials Studio. A 5×5×5 supercell was adopted for MgO, based on an initial structure with lattice parameters *α* = *β* = *γ* = 90°. The lattice parameters *a*, *b*, and *c* were obtained from the experimental data. To calculate the extent of CO_2_ adsorption, the {100} surfaces of MgO were modelled with a vacuum width of 15 Å. A generalized gradient approximation with the Perdew–Burke–Ernzerhof functional was employed to obtain the DFT exchange-correlation energy, and the plane-wave basis set was assigned a cut-off of 490 eV. The self-consistent field tolerance was 2×10^−6^ eV, the Brillouin zone was sampled at 2×2×1 *k*-points, and the core electrons were replaced by ultrasoft pseudopotentials.

All the structural optimizations, charge density difference analysis were carried out by the Vienna Ab initio simulation package (VASP)^[69,70]^ using the DFT method. The generalized gradient approximation (GGA) with the Perdew-Burke-Ernzerhof (PBE)^[71]^ functional was used to describe the electron exchange correlation interactions. In order to overcome the deficiency of GGA, the method of GGA + U was adopted in the calculation process. The value of the effective Hubbard U was set as 5.0 eV for Zn. Calculations were performed with the cutoff plane-wave kinetic energy of 480 eV, and 1×1×1 k-mesh grids were employed for the integration of the Brillouin zone. The convergence criteria for energy and force during geometrical optimization were set to 10^−5^ eV and −0.05 eV/Å, respectively. The vacuum space of 20 Å was applied to avoid the interactions along z-direction. During the optimization, all atoms were allowed to fully relax. A 2×2 supercell of ZnIn_2_S_4_ (001) and 1×2 supercell of MgO(100) were used to model two kinds of heterojunction surfaces.

The following equation (1) was the formula for calculating the adsorption energies (E_ad_),

E_ad_ = E_sm_ − E_s_ − E_m_ (1)

where E_sm_ was the optimized energy of the molecule adsorbed on slab, E_s_ is the energy of the slab, and E_m_ is the energy of the adsorbed molecule.

The Gibbs free-energies (ΔG) of the adsorbed state were determined by equation (2),

ΔG = ΔE + ΔZPE − TΔS (2)

where ΔE is the total energy difference between the reactant and product species adsorbed on catalyst surface obtained directly from DFT calculations, ΔZPE is the change in zero-point energies, T is the temperature (298.15 K), and ΔS is the difference vibrational entropy.

The acid−base strength was described by the reaction of an acid and an indicator (Ind, weak base), as represented by the Hammett acidity function (*H*_0_), equation (3), in which *c* and *γ* denoted the concentration and activity coefficient of indicator, respectively.

*H*_0_ ≡ p*K*_a_ + log (*c*_Ind_/*c*_IndH+_) = −log a_H+_ + log (*γ*_IndH+_/*γ*_Ind_) (3)

Since Hammett indicators are chromophores, the reaction between indicators and surface acidic or basic sites led to a color change of solution. The density of the basic site of catalyst could be calculated with equation (4).

Base density (mmol g^−1^) = ([benzoic acid] × *V*_benzoic acid for titration_) / amount of catalyst (4)

**Evaluation of the photocatalytic activity:**

The photocatalytic activity toward CO_2_ reduction reaction (CO_2_RR) and syngas production was evaluated using a Pyrex reaction cell with a quartz window and a Newport Xe lamp (300 W) equipped with a cutoff filter (λ > 420 nm) and an infrared water filter. The photocatalyst powder (3 mg) was suspended in a mixture of deionized water (2 mL) and acetonitrile (3 mL). Subsequently, CoCl_2_ (1 μmol) and bipyridine (15 mg) were added to form the Co-bpy cocatalyst *in situ*. Triethanolamine (1 mL) was used as the hole scavenger. Prior to performing the photoreduction reaction, the reaction cell was fully purged with CO_2_ gas under dark conditions for 1 h. The evolved gas was analyzed using a gas chromatography (GC) system (Agilent 7890 B) equipped with a flame ionization detector and a thermal conductivity detector. Photocatalytic activity tests, including the photocurrent generation and electrochemical impedance spectroscopy (EIS) measurements, were performed using a potentiostat (IVIUM) and a three-electrode cell containing a 0.1 M Na_2_SO_4_ electrolyte. A Pt wire and a saturated calomel electrode were employed as the counter and reference electrodes, while a Nafion/ethanol/nanocomposite film coated onto fluorine-doped tin oxide glass was used as the working electrode.

**Calculation of the apparent quantum yield (AQY):**

The ZM25 catalyst was dispersed according to the previously described ratio to prepare the photocatalyst reaction system, then reacted for 3 h. Optical bandpass filters were equipped onto the Xe lamp for monochromatic light irradiation. Light intensities were measured using an optical power meter (Newport PMKIT-21-01).

The following equation was used to deduce the AQY: $AQY \left( \% \right)= \frac{(N_{H_{2}}n_{H_{2}}+N_{CO}n_{CO})N_{A}}{\frac{IAt\lambda}{hc}}\times100\%$,

where N_H2_ and N_CO_: Number of electrons required for the formation of H_2_ and CO, n_H2_ and n_CO_: Amount of H_2_ and CO generated in mol, N_A_: Avogadro’s number, I: Intensity of light (W cm^−2^), A: Irradiation area (cm^2^), t: Time of irradiation (s), h: Planck’s constant, and c: Speed of light (m s^−1^).


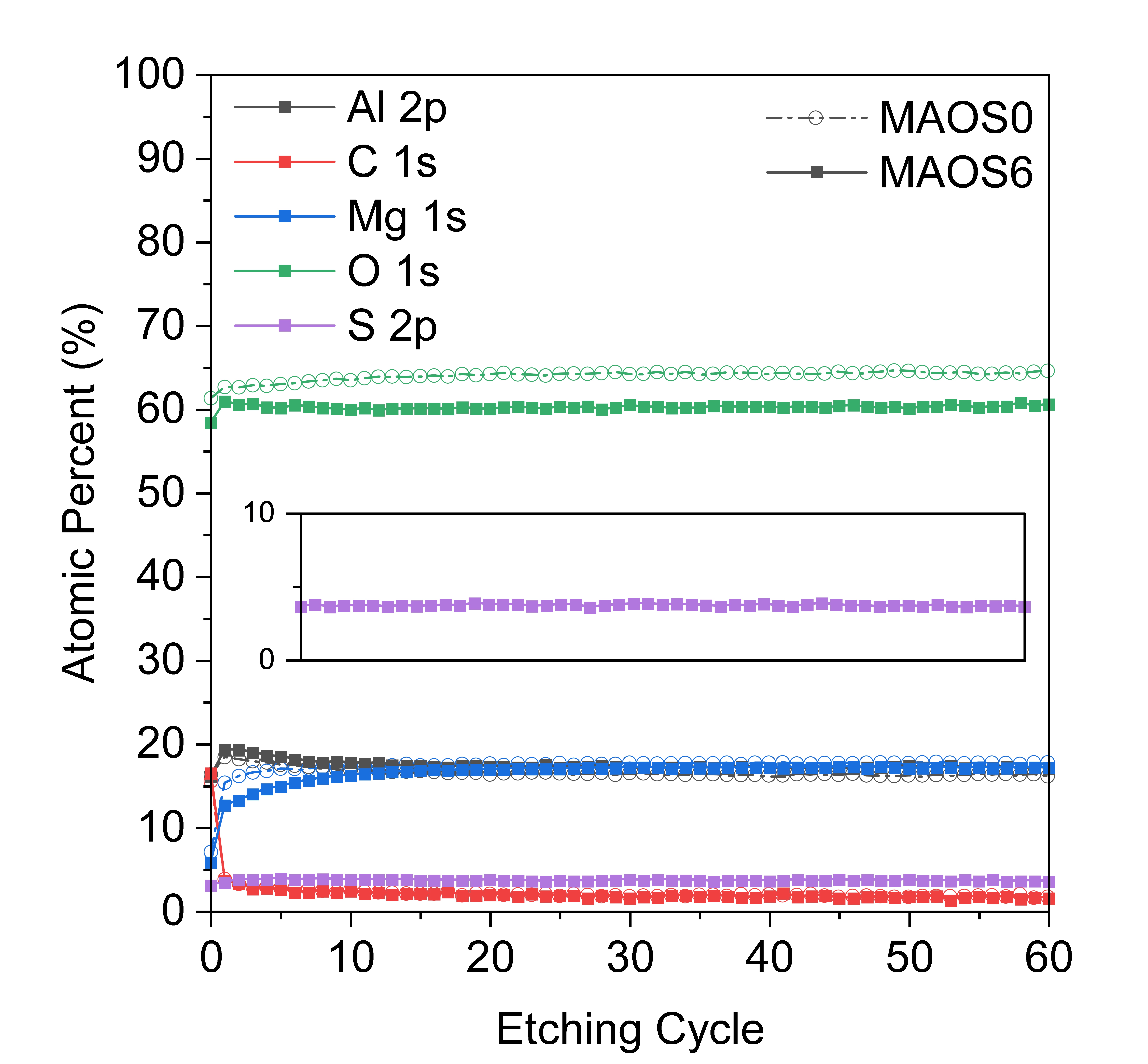


**Figure S1.** XPS depth profile by Ar-ion etching of MAOS6.


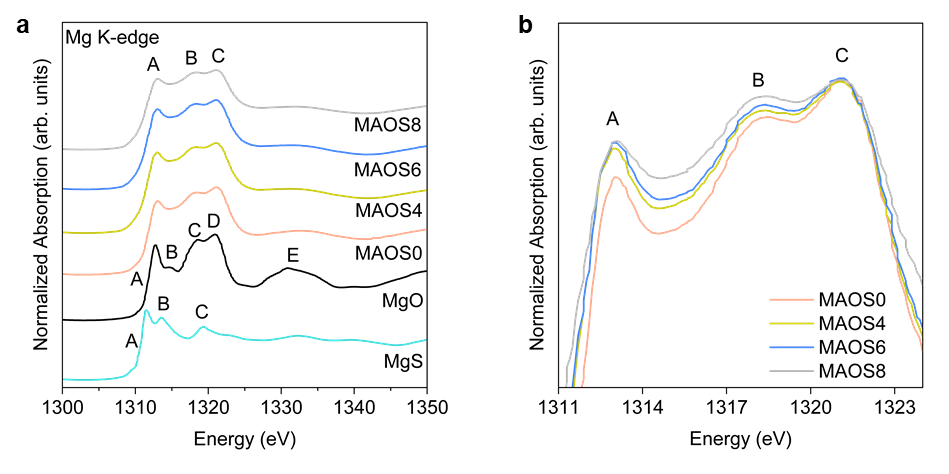


**Figure S2.** a) Mg K-edge XANES spectra and b) the enlarged main-edge spectra of sulfur-doped MAOS nanosheets.


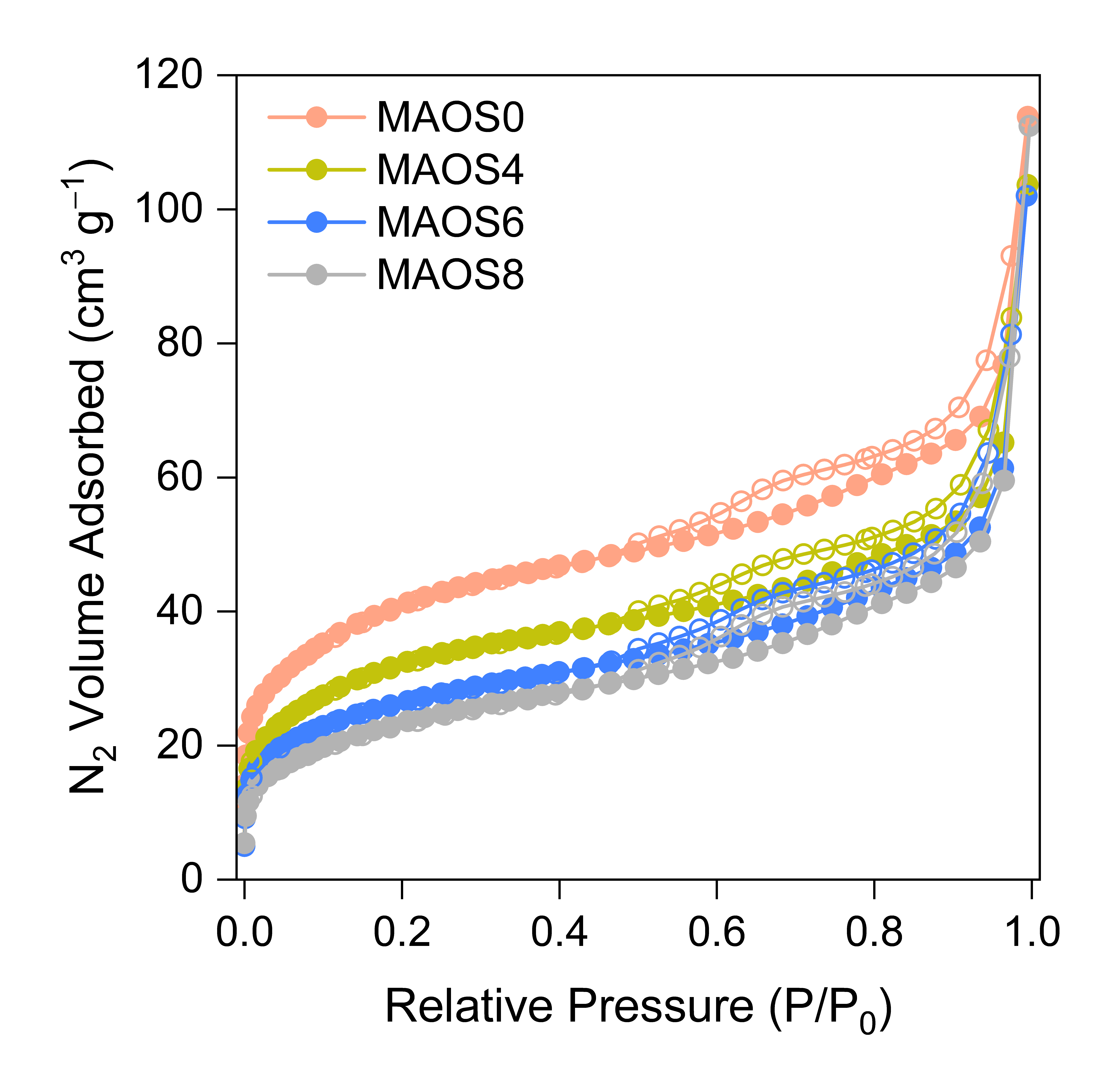


**Figure S3.** N_2_ adsorption−desorption isotherms of MAOS0, MAOS4, MAOS6, and MAOS8 nanosheets.


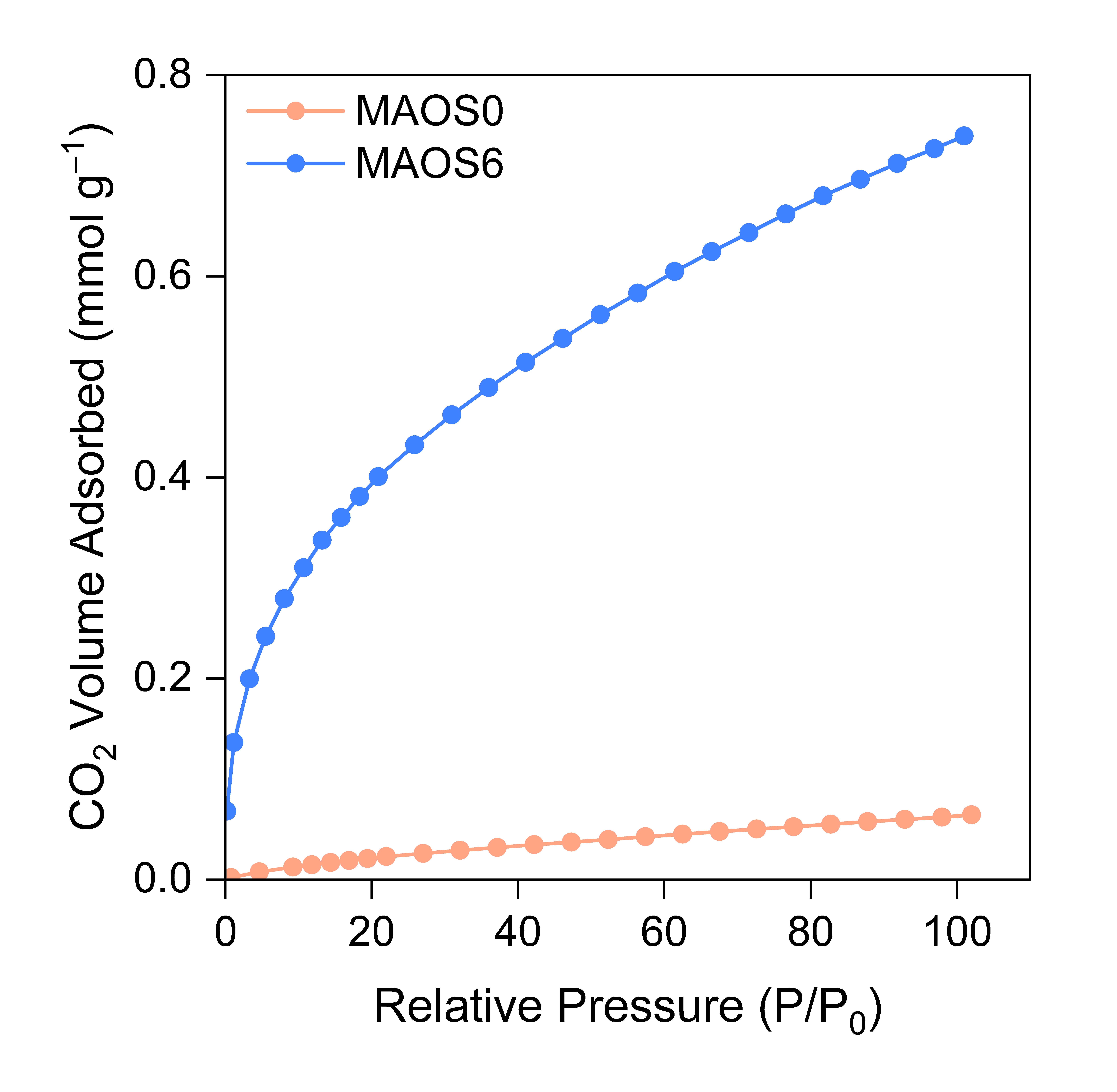


**Figure S4.** CO_2_ adsorption isotherms of MAOS0 and MAOS6 at 0 °C.


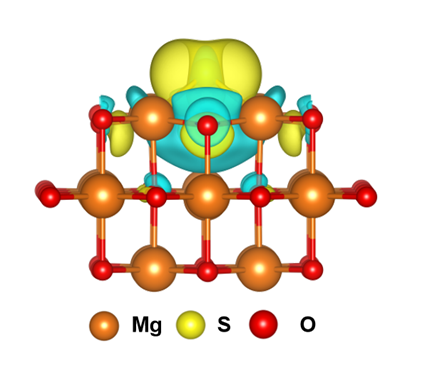


**Figure S5.** Differential charge density of MAOS.


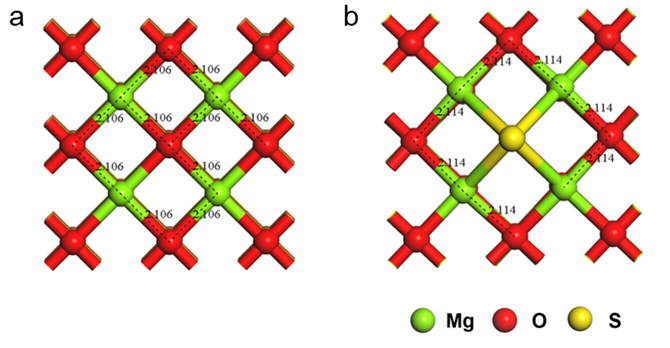


**Figure S6.** The optimized geometry of a) MgO and b) MAOS.

**
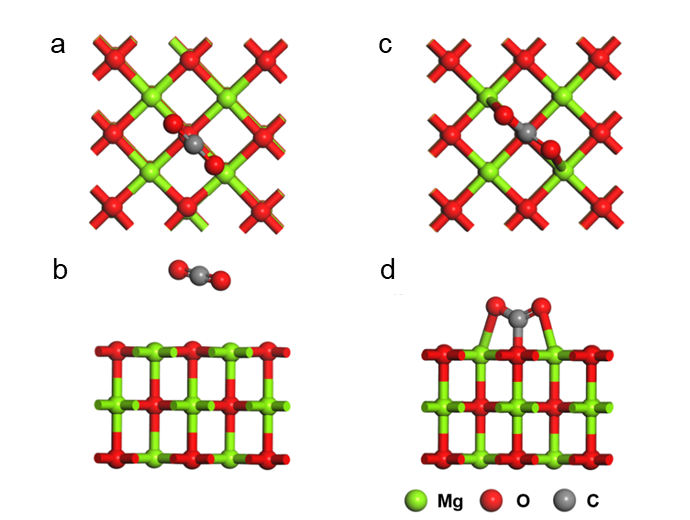
**

**Figure S7.** Supercell model of calculated final geometry of physical adsorbed CO_2_ on the MgO from a) top and b) side view; and chemical adsorbed CO_2_ on the MgO from c) top and d) side view.

**
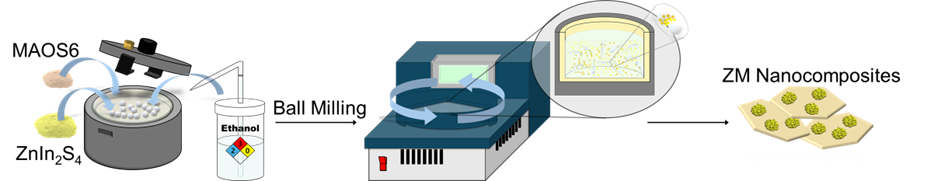
**

**Figure S8.** Schematic illustration for the hybridization between ZIS and MAOS nanosheets.


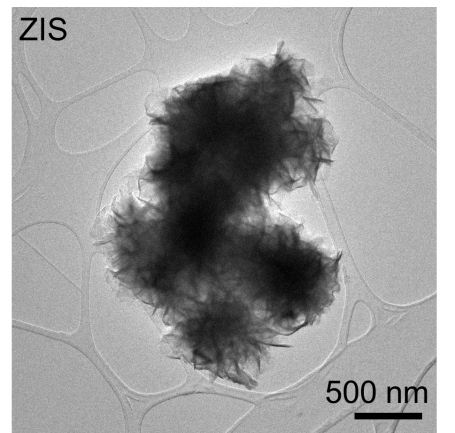


**Figure S9.** TEM image of ZIS.


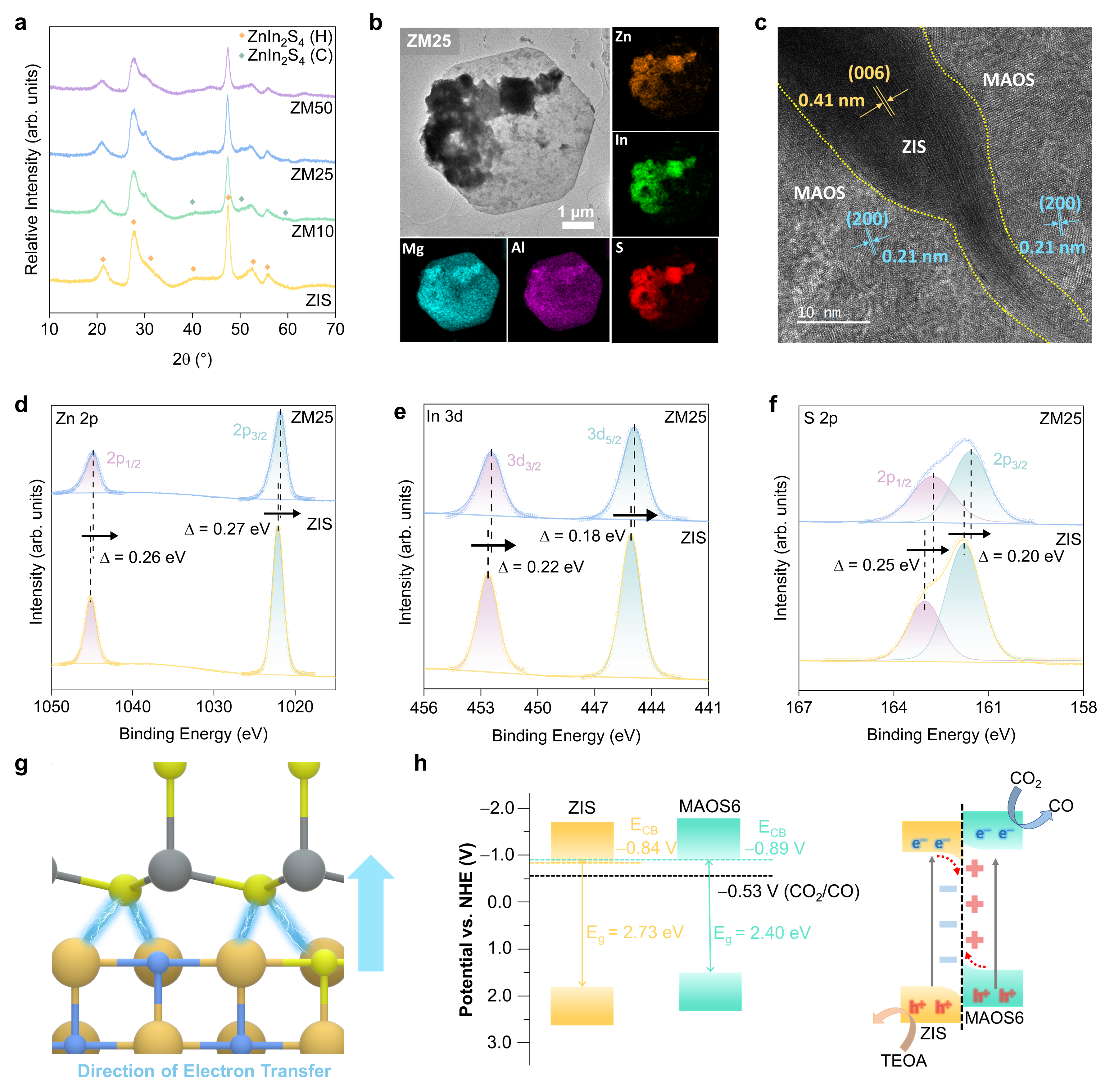


**Figure S10.** HRTEM image of ZM nanocomposite.


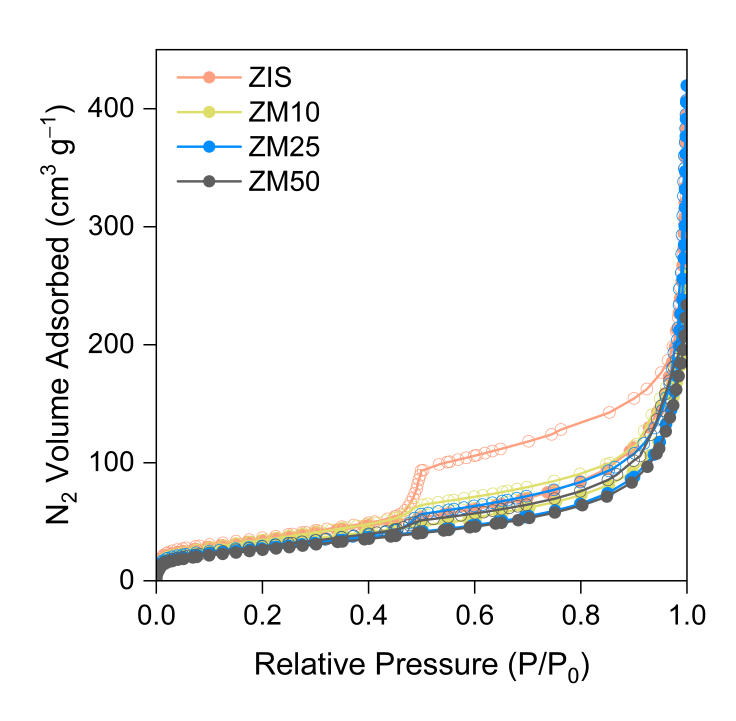


**Figure S11.** N_2_ adsorption−desorption isotherms of ZIS and ZM nanocomposites.


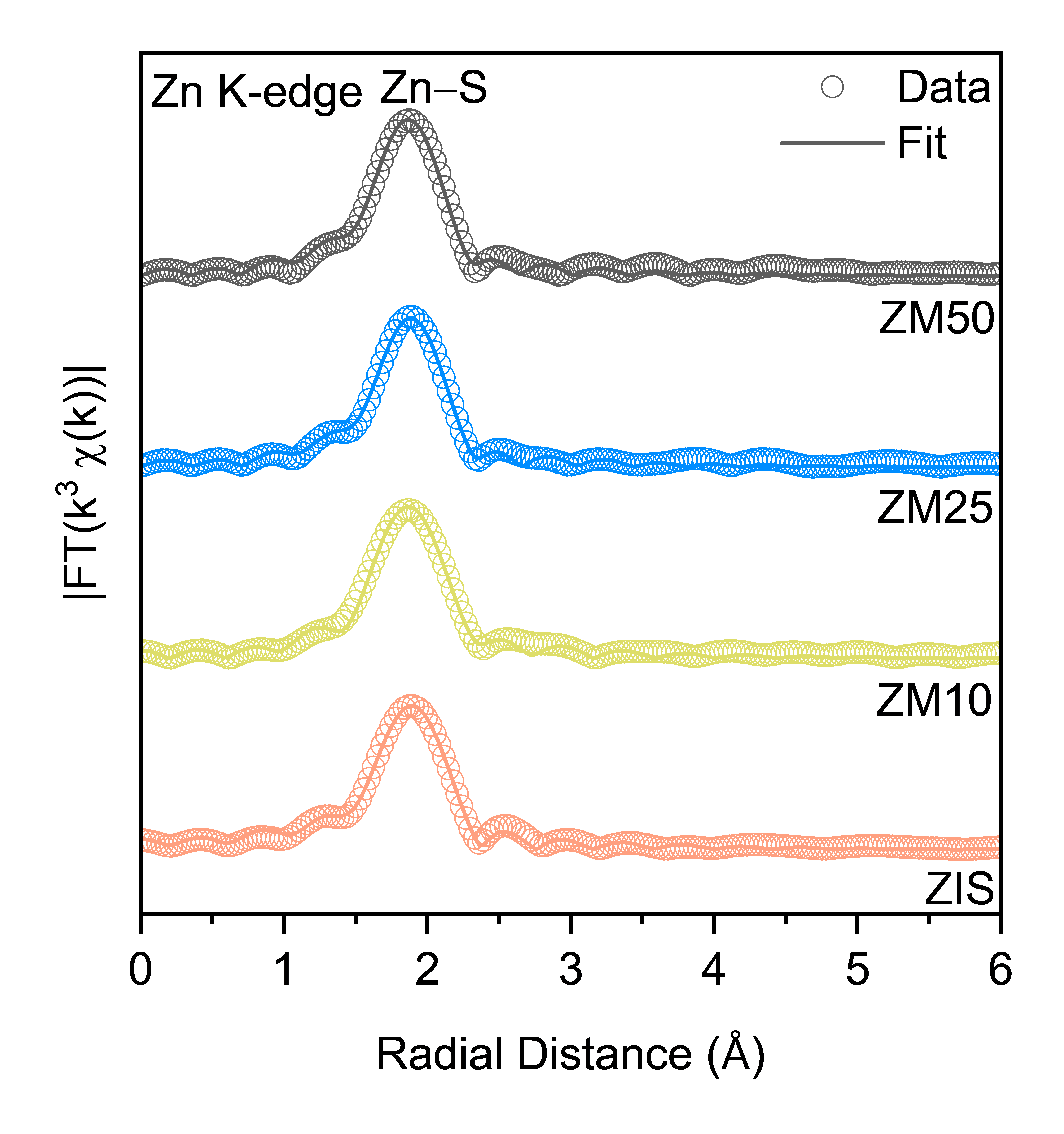


**Figure S12.** Zn K-edge Fourier transformed EXAFS (FT-EXAFS) spectra of ZIS and ZM nanocomposites.


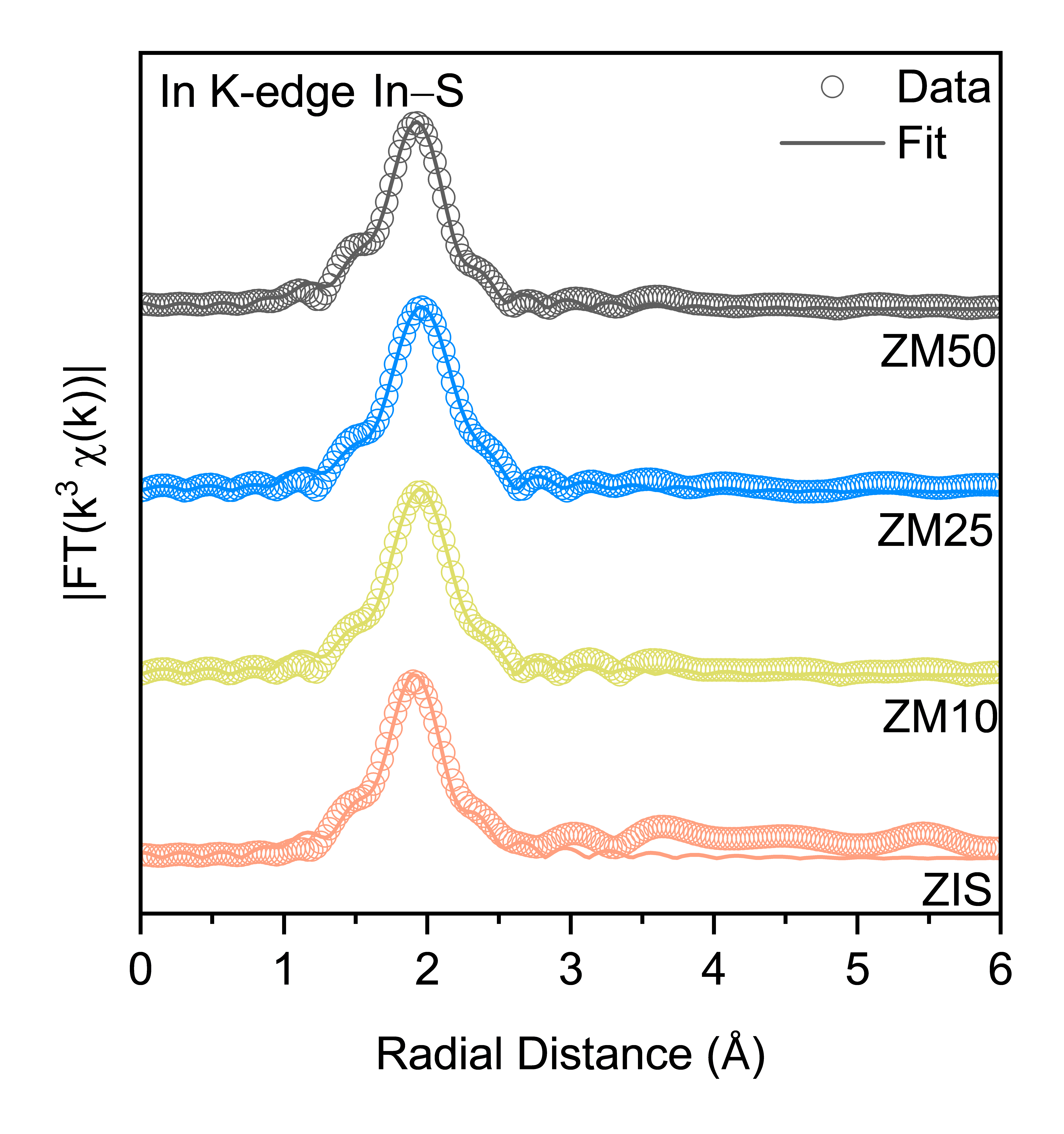


**Figure S13.** In K-edge FT-EXAFS spectra of ZIS and ZM nanocomposites.

**
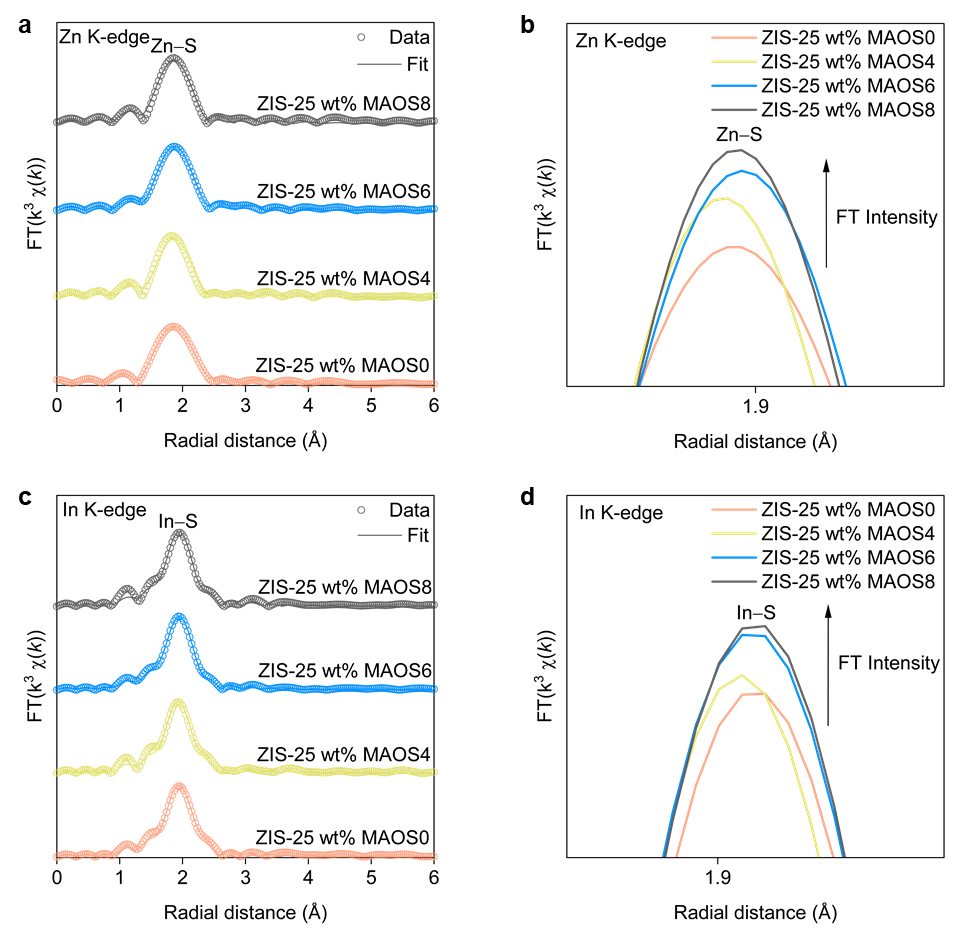
 Figure S14.** a) Zn K-edge FT-EXAFS spectra of ZIS hybridized with 25 wt% MAOS0, MAOS4, MAOS6, and MAOS8, and b) the comparison of the FT intensities. c) In K-edge FT-EXAFS spectra of ZIS hybridized with 25 wt% MAOS0, MAOS4, MAOS6, and MAOS8, and d) the comparison of the FT intensities.


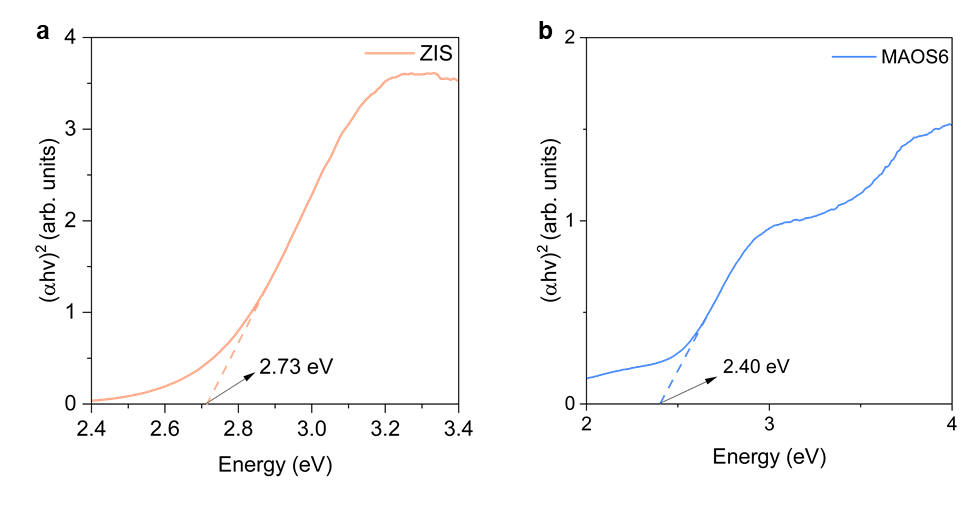


**Figure S15.** Tauc Plots of a) ZIS and b) MAOS6.


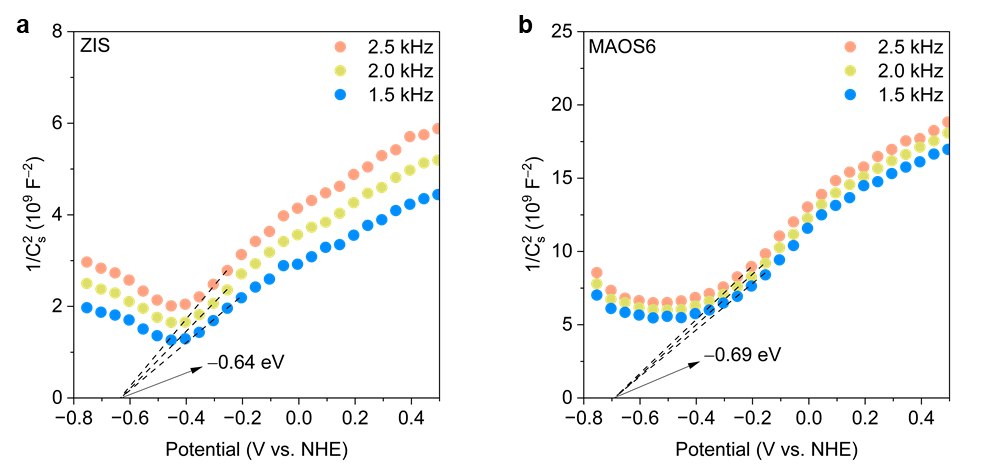


**Figure S16.** Mott−Schottky plots of a) ZIS and b) MAOS6.


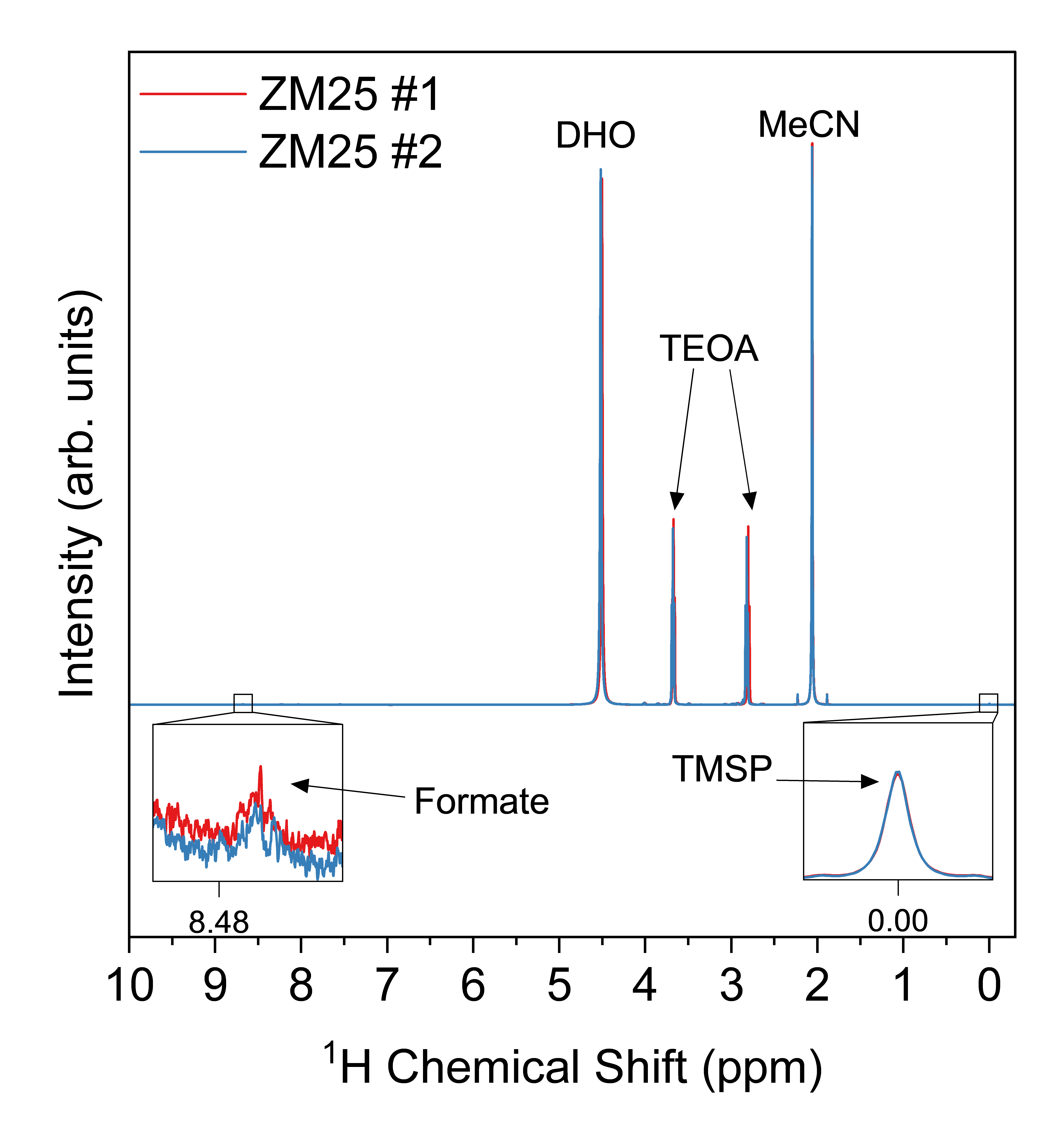


**Figure S17.** ^1^H-nuclear magnetic resonance spectroscopy of the liquid mixture after CO_2_RR.


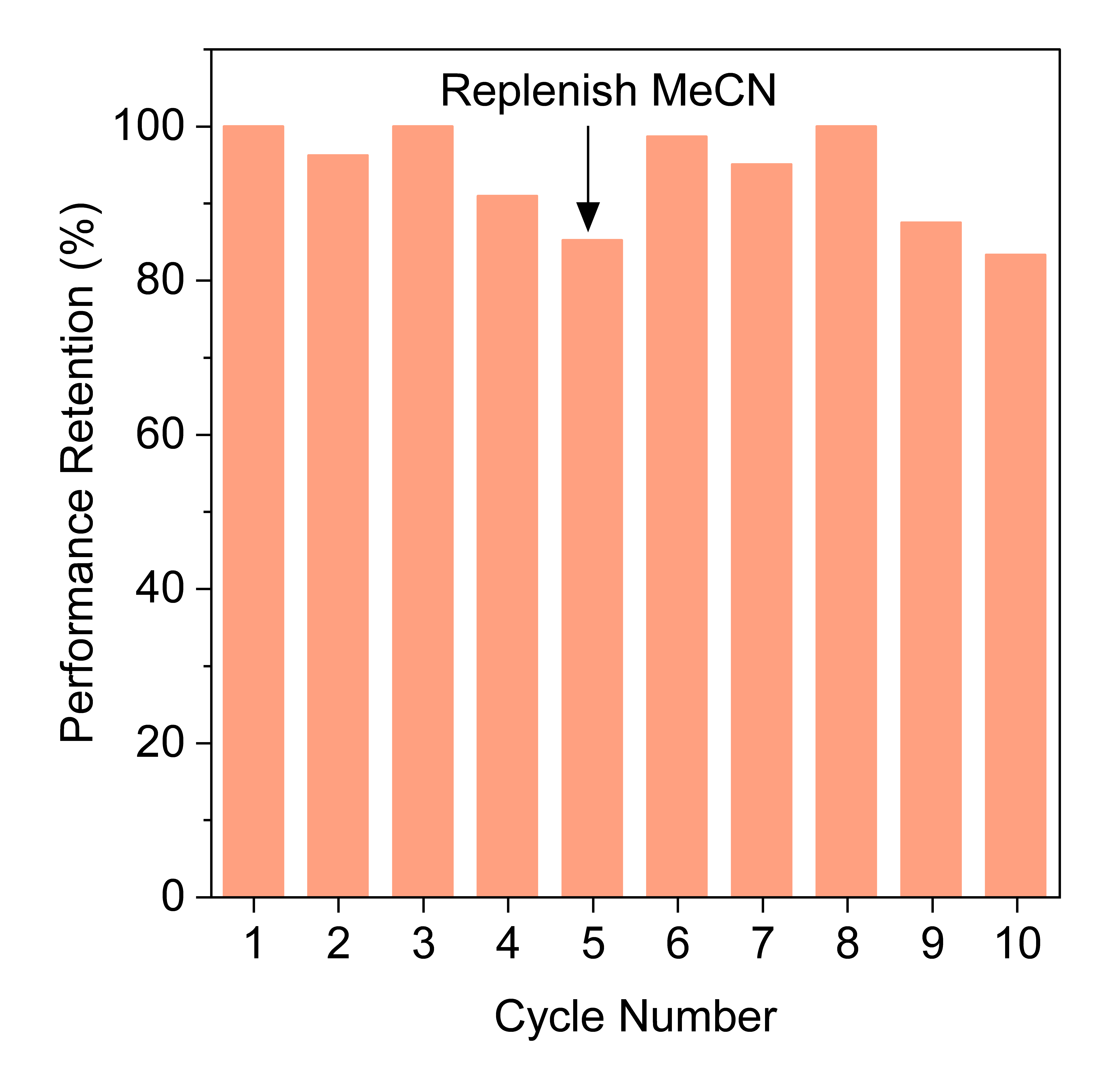


**Figure S18.** Cyclability tests for the photocatalytic activity of ZM25 nanocomposite towards CO_2_RR.

**
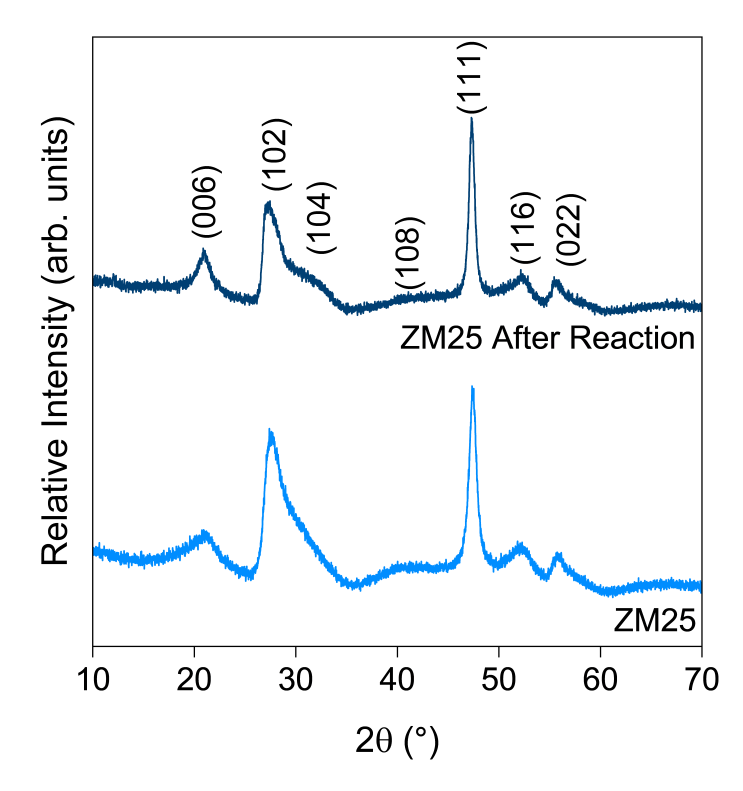
**

**Figure S19.** Powder XRD pattern of the ZM25 nanocomposite before and after the photocatalytic activity test.


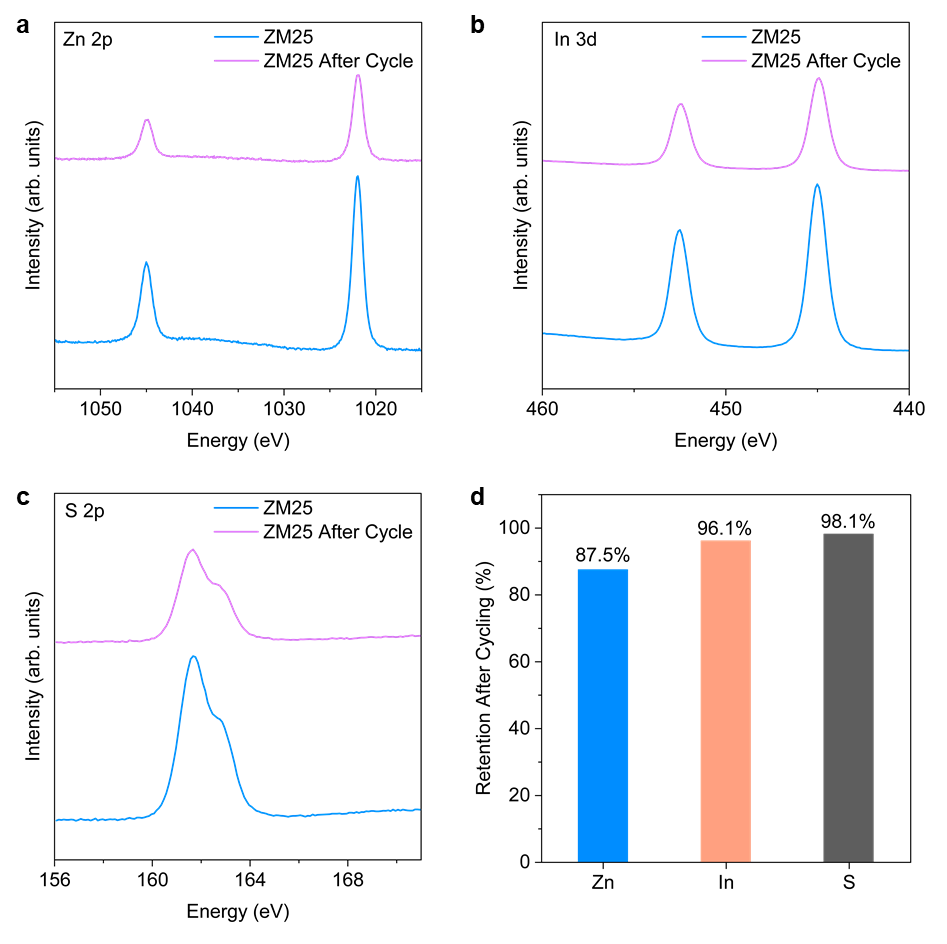


**Figure S20.** a) Zn 2p, b) In 3d, and c) S 2p XPS spectra of ZM25 before and after ten consecutive CO_2_RR cycles. d) Retention of Zn, In, and S after ten consecutive CO_2_RR cycles measured by XPS.

**
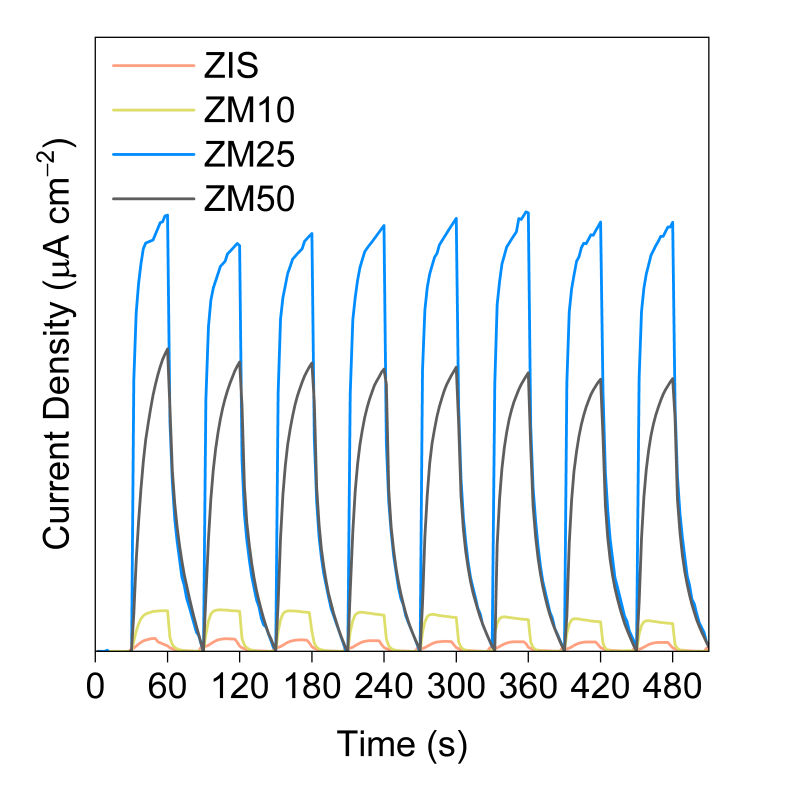
**

**Figure S21.** Photocurrent measurements of ZIS and ZM nanocomposites.


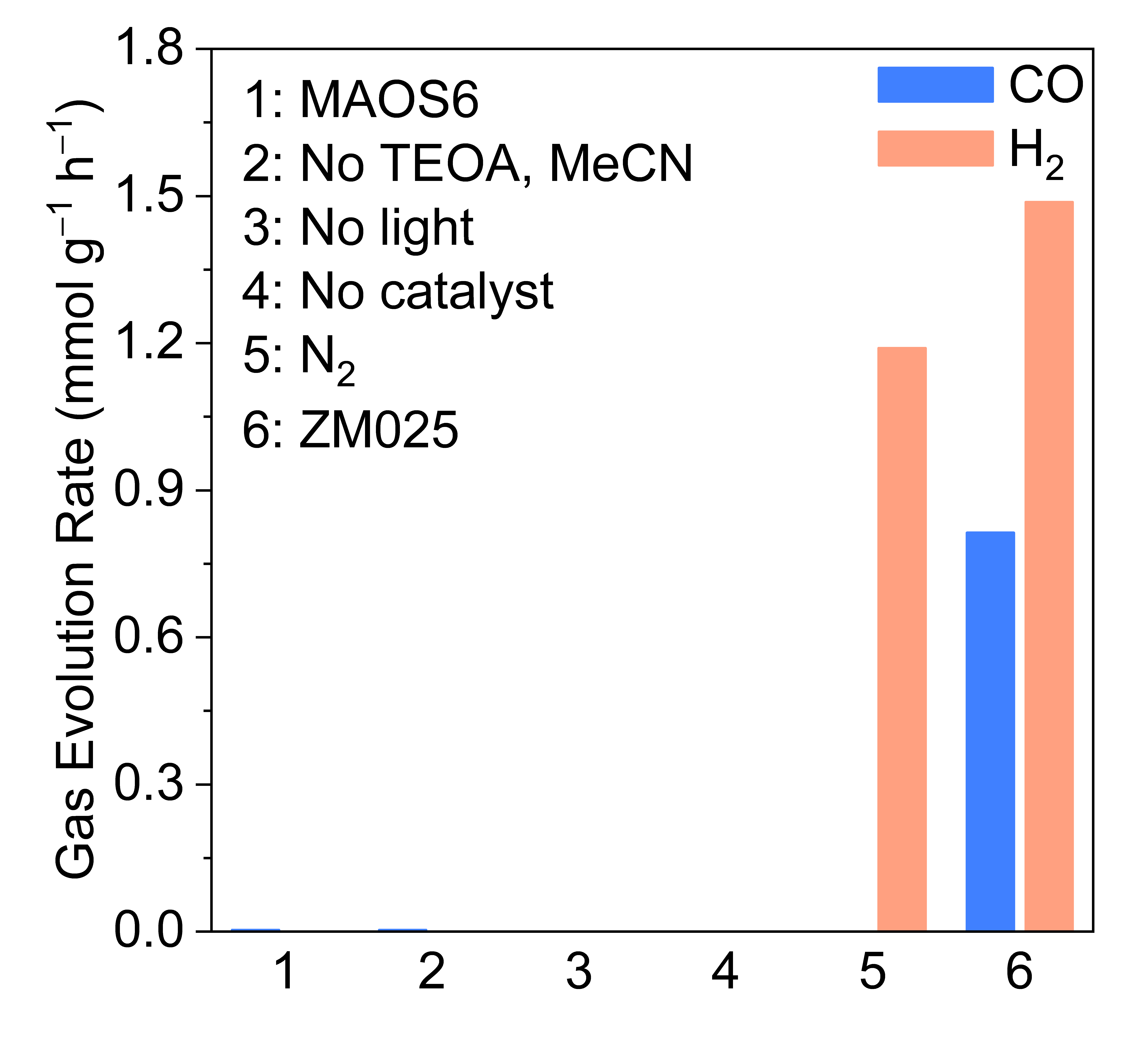


**Figure S22.** Control experiments for photocatalytic activity.


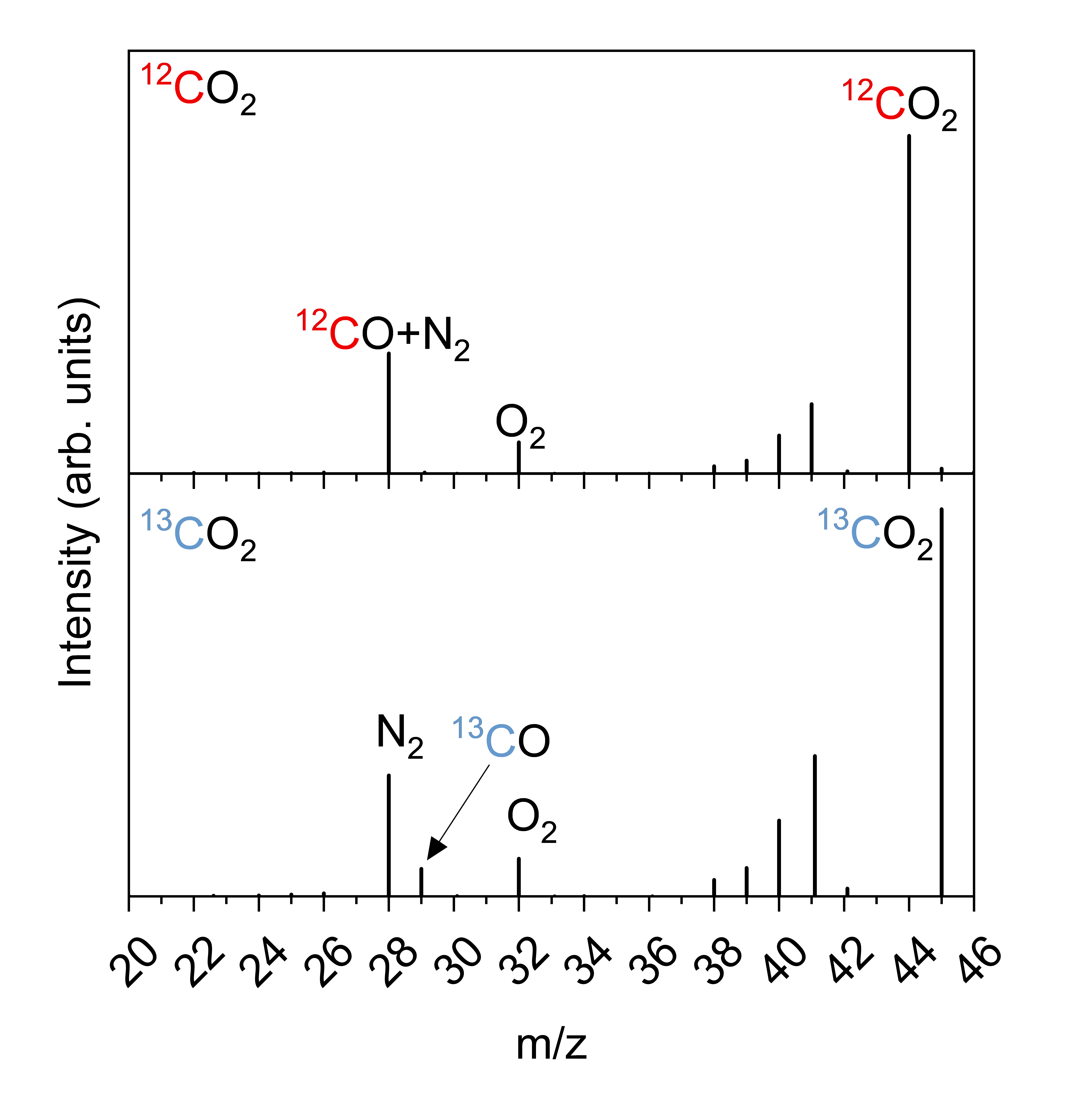


**Figure S23.** GC−mass spectroscopy spectra of the gaseous headspace of the ZM25 reaction system after CO_2_RR with ^12^CO_2_ and ^13^CO_2_ feedstock.

**
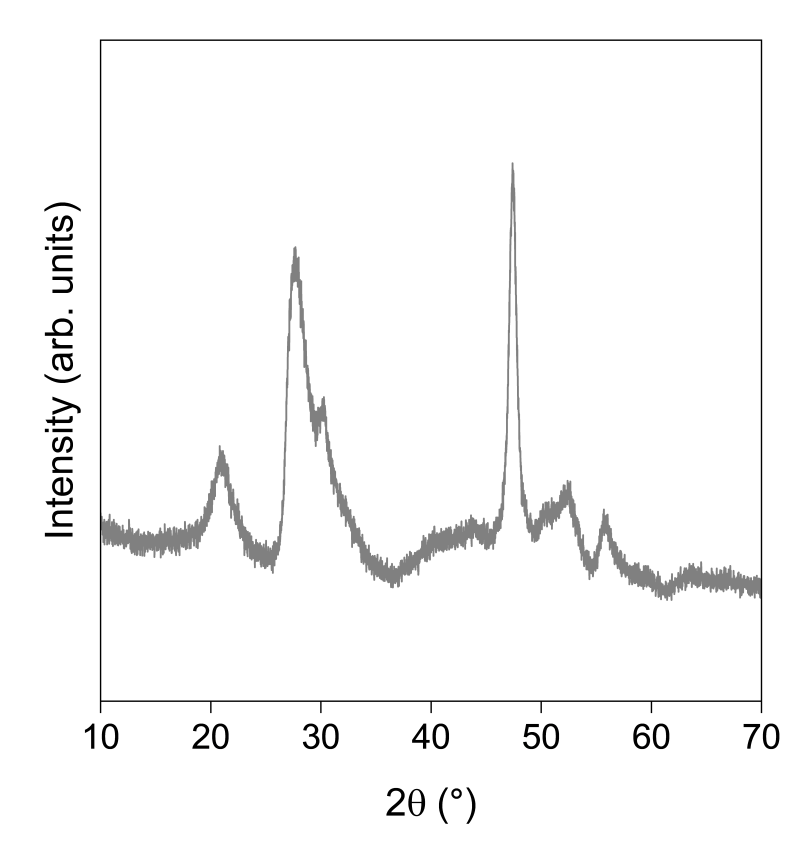
**

**Figure S24.** Powder XRD pattern of ZM025 nanocomposite.

**
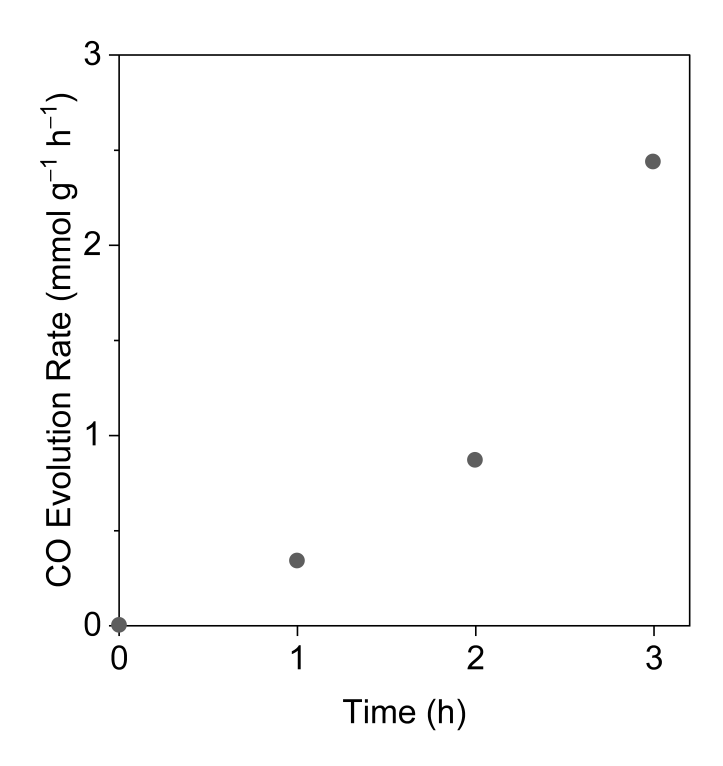
**

**Figure S25.** Photocatalytic CO_2_RR activity of ZM025 nanocomposite.


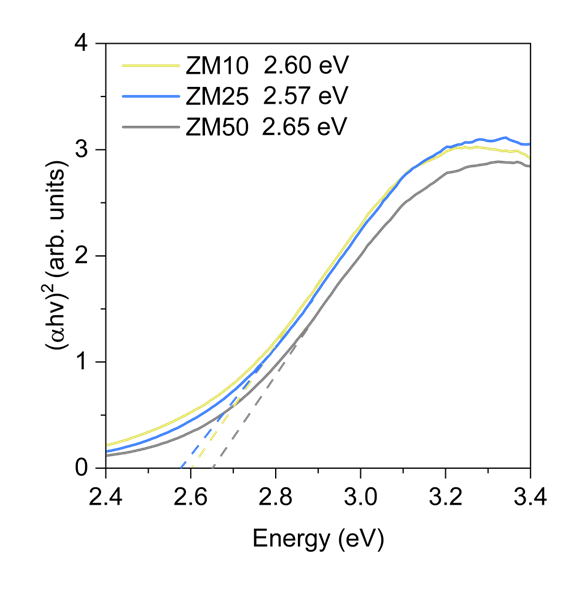


**Figure S26.** Tauc plots of ZM nanocomposites.


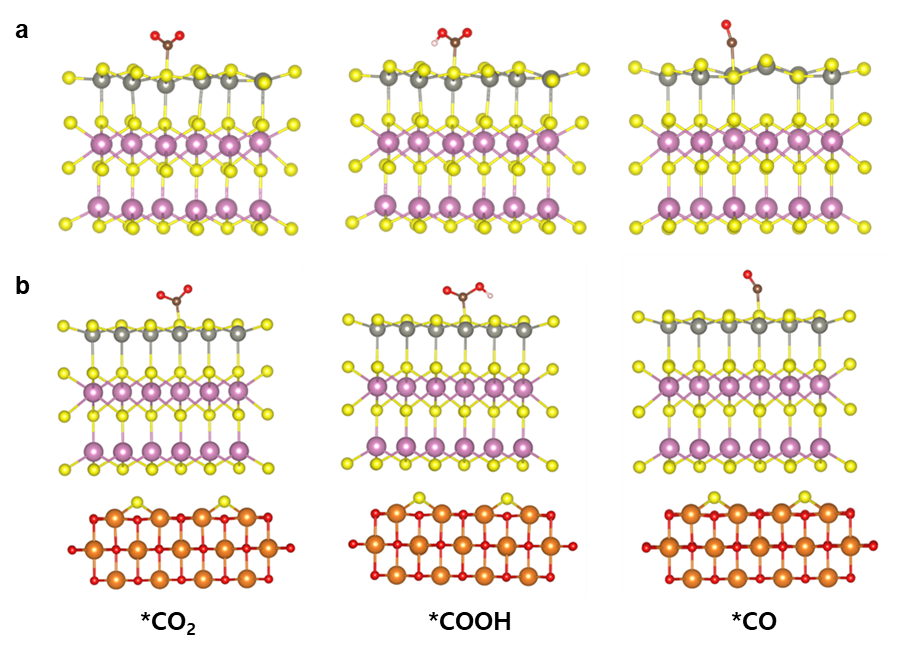


**Figure S27.** DFT models of a) ZIS and b) ZM25.

The MgO (100) surface was selected because it is the most stable and commonly exposed facet of MgO, and the calculated surface energies of MgO (100), MgO (110), and MgO (111) are 1.31, 2.62, and 3.91 J m^−2^, respectively, clearly demonstrating the superior stability of the MgO (100) facet.^[72,73]^ Similarly, the ZnIn_2_S_4_ (001) surface was chosen due to its dominant exposure in the layered nanosheet morphology observed in TEM analysis. As shown in Figure 2b, the ZIS nanoplates were immobilized on the surfaces of larger 2D MAOS6 nanosheets. Therefore, the heterojunction model was constructed by combining ZnIn_2_S_4_ (001) with MgO (100).


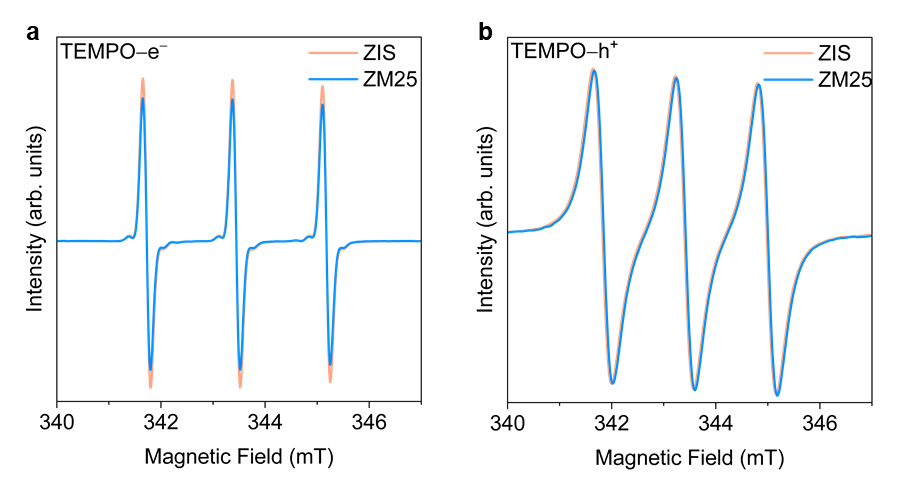


**Figure S28.** *In situ* EPR spectra of ZIS and ZM25 nanocomposite for (a) TEMPO-e^-^ and (b) TEMPO-h^+^.

**Table S1.** Chemical compositions of MAOS nanosheets and Mg−Al-LDH.

| Material | ICP  S/(Mg+Al) | ICP  S/Mg | ICP  Mg/Al |
| --- | --- | --- | --- |
| Mg−Al-LDH |  | - | - |
| MAOS0 | - | - | 1.65 |
| MAOS4 | 0.137 | 0.22 | 1.66 |
| MAOS6 | 0.166 | 0.27 | 1.68 |
| MAOS8 | 0.179 | 0.29 | 1.67 |

**Table S2.** Specific surface areas, pore volumes, and gravimetric and areal CO_2_ adsorption capacities of MAOS nanosheets.

| Material | S_BET_  (m^2^ g^−1^) | Pore volume  (cm^3^ g^−1^) | Gravimetric CO_2_ adsorptivity  (mmol g^−1^) | Areal CO_2_ adsorptivity  (× 10^−3^ mmol m^−2^) |
| --- | --- | --- | --- | --- |
| MAOS0 | 145 | 0.176 | 0.345 | 2.38 |
| MAOS4 | 117 | 0.160 | 0.641 | 5.48 |
| MAOS6 | 94 | 0.158 | 0.779 | 8.29 |
| MAOS8 | 81 | 0.158 | 0.683 | 8.43 |

**Table S3.** CO_2_ adsorption capacity data of Mg−Al-LDH-based materials.

| Material | Temperature (°C) | P_total_ (bar) | CO_2_ adsorption capacity (mmol g^−1^) | Ref |
| --- | --- | --- | --- | --- |
| Mg_3_−Al_1_−CO_3_-LDH | 60 | 1 | 0.319 | 74 |
| Calcined Mg_2_−Al_1_−CO_3_-LDH | 400 | 1 | 0.59 | 75 |
| Mg_2_−Al_1_−CO_3_-LDH/Zn-MOF | 25 | 1 | 0.263 | 76 |
| CHT-10Cs | 300 | 1 | 0.64 | 77 |
| Na/KNO_3_-MG60 | 300 | 1.01 | ~0.75 | 78 |
| cDFM-2−0.4Ni-10Cs | 350 | 1 | 0.48 | 79 |
| Calcined Mg_3_−Al_1_-LDH | 200 | 1 | 0.53 | 80 |
| Calcined Mg_6_−Al_2_−CO_3_-LDH | 400 | 0.8 | 0.58 | 81 |
| MXene/Mg_3_−Al_1_−CO_3_-LDH | 25 | 1 | 0.517 | 82 |
| Sulfur-doped Mg−Al-LDO  (MAOS6) | 300 | 1 | 0.779 | This  work |

**Table S4.** Base density of MAOS nanosheets.

| Material | Base density (mmol g^−1^) |
| --- | --- |
| MAOS0 | 0.15 |
| MAOS4 | 0.32 |
| MAOS6 | 0.41 |
| MAOS8 | 0.42 |

**Table S5.** Bader charge analysis of MgO and MAOS.

| Material | O (e) | Mg (e) |
| --- | --- | --- |
| MgO | +1.65 | −1.65 |
| MAOS | +1.67 | −1.66 |

* "+" represents obtaining electrons and "−" represents electron loss.

**Table S6.** Zn K-edge EXAFS fitting results of the ZM nanocomposites and ZIS.

| Material | Bonding pair | CN | R (Å) | σ^2^ (10^−3^×Å^2^) | ΔE (eV) | R factor |
| --- | --- | --- | --- | --- | --- | --- |
| ZIS^[a]^ | Zn−S | 4.0* | 2.34 | 6.91 | −0.90 | 0.01 |
| ZM10^[b]^ | Zn−S | 4.1 | 2.34 | 6.72 | −0.01 | 0.01 |
| ZM25^[c]^ | Zn−S | 4.2 | 2.35 | 7.81 | 0.15 | 0.01 |
| ZM50^[d]^ | Zn−S | 4.3 | 2.34 | 7.33 | −1.41 | 0.01 |

* In the course of the fitting analysis for ZIS, the coordination number of the Zn−S shell was fixed to determine the amplitude reduction factor.

The curve fitting analysis was performed for the range of ^[a]^1.30-R-3.00 Å and 3.521-k-11.040 Å^−1^; ^[b]^1.35-R-3.00 Å and 2.643-k-10.107 Å^−1^; ^[c]^1.30-R-2.95 Å and 2.632-k-10.986 Å^−1^; ^[d]^1.35-R-3.00 Å and 2.659-k-11.013 Å^−1^.

**Table S7.** In K-edge EXAFS fitting results of the ZM nanocomposites and ZIS.

| Material | Bonding pair | CN | R (Å) | σ^2^ (10^−3^×Å^2^) | ΔE (eV) | R factor |
| --- | --- | --- | --- | --- | --- | --- |
| ZIS^[a]^ | In−S | 4.0* | 2.44 | 3.62 | 0.20  0.20 | 0.03 |
|  | In−S | 6.0* | 2.59 | 7.11 |  | 0.03 |
| ZM10^[b]^ | In−S | 4.1 | 2.45 | 4.06 | 4.62  4.62 | 0.01 |
|  | In−S | 6.2 | 2.60 | 8.01 |  | 0.01 |
| ZM25^[c]^ | In−S | 4.2 | 2.45 | 4.36 | 5.89  5.89 | 0.01 |
|  | In−S | 6.2 | 2.60 | 7.95 |  | 0.01 |
| ZM50^[d]^ | In−S | 4.2 | 2.45 | 4.34 | 0.89  0.89 | 0.01 |
|  | In−S | 6.3 | 2.60 | 8.27 |  | 0.01 |

* In the course of the fitting analysis for ZIS, the coordination number of the In−S shell was fixed to determine the
amplitude reduction factor.

The curve fitting analysis was performed for the range of ^[a]^1.30-R-3.00 Å and 2.470-k-13.169 Å^−1^; ^[b]^1.40-R-2.95 Å and 2.686-k-12.387 Å^−1^; ^[c]^1.40-R-3.00 Å and 2.712-k-12.414 Å^−1^; ^[d]^1.30-R-3.00 Å and 2.362-k-13.142 Å^−1^.

**Table S8.** Summary of ionic radii, covalent radii, and van der Waals radii of Zn, In, and S.

| Ionic Radius (Å) | Covalent Radius (Å) | van der Waals Radius (Å) |
| --- | --- | --- |
| Zn^2+^ (T_d_): 0.60 | Zn: 1.22  In: 1.42  S: 1.05 | Zn: 1.39  In: 1.93  S: 1.80 |
| In^3+^ (T_d_): 0.62 |  |  |
| In^3+^ (O_h_): 0.80 |  |  |
| S^2−^: 1.84 |  |  |

**Table S9.** Comparison of Zn−S bond lengths.

| Material Name | Sum of Ionic Radii (Å) | Sum of Covalent Radii (Å) | Sum of van der Waals Radii (Å) | Bond Length by EXAFS Fitting (Å) |
| --- | --- | --- | --- | --- |
| ZIS-25 wt% MAOS0 | 2.44 | 2.27 | 3.19 | 2.34 |
| ZIS-25 wt% MAOS4 |  |  |  | 2.34 |
| ZIS-25 wt% MAOS6 |  |  |  | 2.34 |
| ZIS-25 wt% MAOS8 |  |  |  | 2.34 |

**Table S10.** Comparison of In−S bond lengths.

| Material Name | Sum of Ionic Radii (Å) | Sum of Covalent Radii (Å) | Sum of van der Waals Radii (Å) | Bond Length by EXAFS Fitting (Å) |
| --- | --- | --- | --- | --- |
| ZIS-25 wt% MAOS0 | T_d_: 2.46  O_h_: 2.64 | 2.47 | 3.73 | T_d_: 2.45  O_h_: 2.60 |
| ZIS-25 wt% MAOS4 |  |  |  | T_d_: 2.45  O_h_: 2.60 |
| ZIS-25 wt% MAOS6 |  |  |  | T_d_: 2.45  O_h_: 2.59 |
| ZIS-25 wt% MAOS8 |  |  |  | T_d_: 2.46  O_h_: 2.60 |

**Table S11.** Comparison of photocatalytic syngas production performances to recently published reports.

| Material | Syngas Production Rate  (μmol g^−1^ h^−1^) | CO/H_2_ Ratio | Measurement Conditions | Ref |
| --- | --- | --- | --- | --- |
| Se-ZIS | 1991.1 | 1.5 | 4 mg catalyst, 0.2 µmol CoCl_2_,  15 mg bpy, 3 mL MeCN,  1 mL TEOA, 2 mL H_2_O. | 56 |
| COF/ZIS | 2707.7 | 1.2 | 5 mg catalyst, 1.0 µmol CoCl_2_,  15 mg bpy, 3 mL MeCN,  1 mL TEOA, 1 mL H_2_O. | 57 |
| CdIn_2_S_4_/ZIS | 1670.2 | 2.5 | 10 mg catalyst, 25 mL of  (0.154 g CoCl_2_⋅6H_2_O, 1 g bpy,  200 mL MeCN, 100 mL TEOA,  140 mL 0.5 M KHCO_3_). | 58 |
| CeO_2_/g-C­_3_N_4_ | 1598.68 | 2.7 | 1 mg catalyst,  7.5 mg [Ru(bpy)_3_]Cl_2_⋅6H_2_O,  3 mL MeCN, 1 mL TEOA,  1 mL H_2_O. | 59 |
| ZIS/g-C_3_N_4_ | 3650 | 1.4 | 10 mg catalyst, 2.0 µmol CoCl_2_, 15 mg bpy, 9 mL MeCN,  2 mL TEOA, 1 mL H_2_O. | 60 |
| CoSi_2_/Si | 3000 | 0.8 | 2 mg catalyst,  4 mg [Ru(bpy)_3_]Cl_2_⋅6H_2_O,  4 mL MeCN, 1 mL TEOA. | 61 |
| M-Salen Zr-MOF | 3440 | 1.3 | 10 mg catalyst,  60 mg [Ru(bpy)_3_]Cl_2_⋅6H_2_O,  70 mL MeCN, 20 mL TEOA,  10 mL H_2_O. | 62 |
| Cu-In_2_S_3_ | 205.3 | 0.9 | 30 mg catalyst,  5 mg [Ru(bpy)_3_]Cl_2_⋅6H_2_O,  3 mL MeCN, 1 mL TEOA,  2 mL H_2_O. | 63 |
| ZM25 | 3299 | 2.2 | 3 mg catalyst, 1 µmol CoCl_2_,  15 mg bpy, 3 mL MeCN,  1 mL TEOA, 2 mL H_2_O. | This work |

**Table S12.** The calculated zero-point energy and entropy of different adsorbed species on the MgOS.

| Adsorbed species | E (eV) | ZPE (eV) | TS (eV) |
| --- | --- | --- | --- |
| *CO_2_ | −155.35 | 0.305 | 0.011 |
| *COOH | −158.15 | 0.634 | 0.098 |
| *CO | −147.15 | 0.139 | 0.118 |

**Table S13.** The calculated zero-point energy and entropy of different adsorbed species on the ZIS.

| Adsorbed species | E (eV) | ZPE (eV) | TS (eV) |
| --- | --- | --- | --- |
| *CO_2_ | −181.73 | 0.304 | 0.074 |
| *COOH | −185.74 | 0.610 | 0.127 |
| *CO | −173.69 | 0.141 | 0.098 |

**Table S14.** The calculated zero-point energy and entropy of different adsorbed species on the ZM nanocomposite.

| Adsorbed species | E (eV) | ZPE (eV) | TS (eV) |
| --- | --- | --- | --- |
| *CO_2_ | −449.26 | 0.351 | 0.147 |
| *COOH | −453.36 | 0.686 | 0.250 |
| *CO | −441.93 | 0.253 | 0.068 |

**Table S15.** The dissociation energy of water on ZIS and ZM25.

| Adsorbed species | E_H2O_ (eV) | E_OH+H_ (eV) | ∆E (eV) |
| --- | --- | --- | --- |
| ZIS | −173.34 | −172.20 | 1.13 |
| ZM | −439.55 | −439.40 | 0.15 |

**Table S16.** *In situ* Zn K-edge EXAFS fitting results of ZIS and ZM25 under light-on and light-off conditions.

| Material | Bonding pair | CN | R (Å) | σ^2^ (10^−3^×Å^2^) | ΔE (eV) | R factor |
| --- | --- | --- | --- | --- | --- | --- |
| ZIS^[a]^ | Zn−S | 4.0* | 2.34 | 6.97 | 1.77 | 0.01 |
| ZIS (On)^[b]^ | Zn−S | 3.9 | 2.34 | 6.98 | 2.30 | 0.01 |
| ZIS (Off)^[c]^ | Zn−S | 4.0 | 2.34 | 7.06 | 1.96 | 0.01 |
| ZM25^[d]^ | Zn−S | 4.2 | 2.34 | 7.64 | 2.45 | 0.01 |
| ZM25 (On)^[e]^ | Zn−S | 3.8 | 2.34 | 6.96 | 2.09 | 0.01 |
| ZM25 (Off)^[f]^ | Zn−S | 4.0 | 2.34 | 7.31 | 2.30 | 0.01 |

* In the course of the fitting analysis for ZIS, the coordination number of the Zn−S shell was fixed to determine the amplitude reduction factor.

The curve fitting analysis was performed for the range of ^[a]^1.40-R-3.00 Å and 2.760-k-10.242 Å^−1^; ^[b]^1.45-R-3.00 Å and 2.778-k-10.265 Å^−1^; ^[c]^1.45-R-2.95 Å and 2.756-k-10.218 Å^−1^; ^[d]^1.35-R-2.95 Å and 2.731-k-10.922 Å^−1^; ^[e]^1.30-R-2.90 Å and 2.724-k-10.898 Å^−1^; ^[d]^1.30-R-3.00 Å and 2.742-k-10.945 Å^−1^.

**Table S17.** *In situ* In K-edge EXAFS fitting results of ZIS and ZM25 under light-on and light-off conditions.

| Material | Bonding pair | CN | R (Å) | σ^2^ (10^−3^×Å^2^) | ΔE (eV) | R factor |
| --- | --- | --- | --- | --- | --- | --- |
| ZIS^[a]^ | In−S | 4.0* | 2.45 | 4.16 | 3.64  3.64 | 0.01 |
|  | In−S | 6.0* | 2.60 | 6.91 |  | 0.01 |
| ZIS (On)^[b]^ | In−S | 3.9 | 2.44 | 4.26 | 4.26  4.26 | 0.01 |
|  | In−S | 5.9 | 2.59 | 6.81 |  | 0.01 |
| ZIS (Off)^[c]^ | In−S | 4.0 | 2.45 | 4.26 | 1.96  1.96 | 0.01 |
|  | In−S | 5.9 | 2.60 | 7.07 |  | 0.01 |
| ZM25^[d]^ | In−S | 4.2 | 2.45 | 5.18 | 3.93 | 0.01 |
|  | In−S | 6.3 | 2.60 | 9.05 | 3.93 | 0.01 |
| ZM25 (On)^[e]^ | In−S | 4.0 | 2.45 | 4.92 | 2.23 | 0.01 |
|  | In−S | 6.1 | 2.60 | 8.72 | 2.23 | 0.01 |
| ZM25 (Off)^[f]^ | In−S | 4.1 | 2.45 | 5.10 | 1.90  1.90 | 0.01 |
|  | In−S | 6.1 | 2.60 | 8.11 |  | 0.01 |

* In the course of the fitting analysis for ZIS, the coordination number of the In−S shell was fixed to determine the amplitude reduction factor.

The curve fitting analysis was performed for the range of ^[a]^1.35-R-3.00 Å and 2.470-k-10.797 Å^−1^; ^[b]^1.30-R-3.00 Å and 2.515-k-10.859 Å^−1^; ^[c]^1.35-R-2.95 Å and 2.474-k-11.448 Å^−1^; ^[d]^1.30-R-3.00 Å and 2.443-k-10.851 Å^−1^; ^[e]^1.35-R-2.95 Å and 2.389-k-11.488 Å^−1^; ^[d]^1.35-R-3.00 Å and 2.389-k-11.434 Å^−1^.
